# Photocatalysis with organic dyes: facile access to reactive intermediates for synthesis

**DOI:** 10.3762/bjoc.16.103

**Published:** 2020-05-29

**Authors:** Stephanie G E Amos, Marion Garreau, Luca Buzzetti, Jerome Waser

**Affiliations:** 1 Laboratory of Catalysis and Organic Synthesis, Ecole Polytechnique Fédérale de Lausanne, EPFL, SB ISIC LCSO, BCH 4306 1015 Lausanne, Switzerland

**Keywords:** organic dyes, photocatalysis, photoredox catalysis, radicals, reactive intermediates

## Abstract

Organic dyes have emerged as a reliable class of photoredox catalysts. Their great structural variety combined with the easy fine-tuning of their electronic properties has unlocked new possibilities for the generation of reactive intermediates. In this review, we provide an overview of the available approaches to access reactive intermediates that employ organophotocatalysis. Our contribution is not a comprehensive description of the work in the area but rather focuses on key concepts, accompanied by a few selected illustrative examples. The review is organized along the type of reactive intermediates formed in the reaction, including C(sp^3^) and C(sp*^2^*) carbon-, nitrogen-, oxygen-, and sulfur-centered radicals, open-shell charged species, and sensitized organic compounds.

## Review

### Introduction

In the last decade, synthetic organic chemistry has experienced the renaissance of photocatalysis. Since the early seminal reports [1–4], inspired by pioneering works in photochemistry [5–7], this field has attracted increasing attention, and organic chemists have developed a wide variety of photocatalytic reactions [8–11]. One of the reasons for this rapid growth resides in the availability of visible light-absorbing transition metal complexes. These catalysts can harvest the energy of visible-light photons and transfer it to organic molecules, giving access to key reactive intermediates. For instance, ruthenium and iridium polypyridyl complexes played a central role in the rapid expansion of photocatalytic methods [12]. These catalysts typically absorb light in the blue region and promote different activation modes, including photoinduced electron transfer (PET) and energy transfer (EnT), which respectively lead to the formation of open-shell and electronically excited species. These reactive intermediates are then used to forge new chemical bonds or to induce structural modifications within the organic substrates. The versatility of these metal complexes is due to their wide operational redox windows, which allows them to interact via their excited states with different classes of molecules. However, the relatively high cost of these photocatalysts, their toxicity, and the limited abundance of the coordinating transition metals can hamper their applicability [13]. For these reasons, the quest for cheaper, more sustainable, and environmentally benign photocatalysts is of high importance, and organic dyes represent a powerful platform for pursuing this goal.

The ability of organic dyes to absorb light and promote transformations is known since the early stage of photochemistry, and they represent attractive alternatives to the established transition metal-based photocatalysts [14–18]. In addition to their ready availability and low cost, these molecules are often biocompatible, and they can be easily functionalized in order to modulate their spectroscopical and redox features.

**Figure 1:**
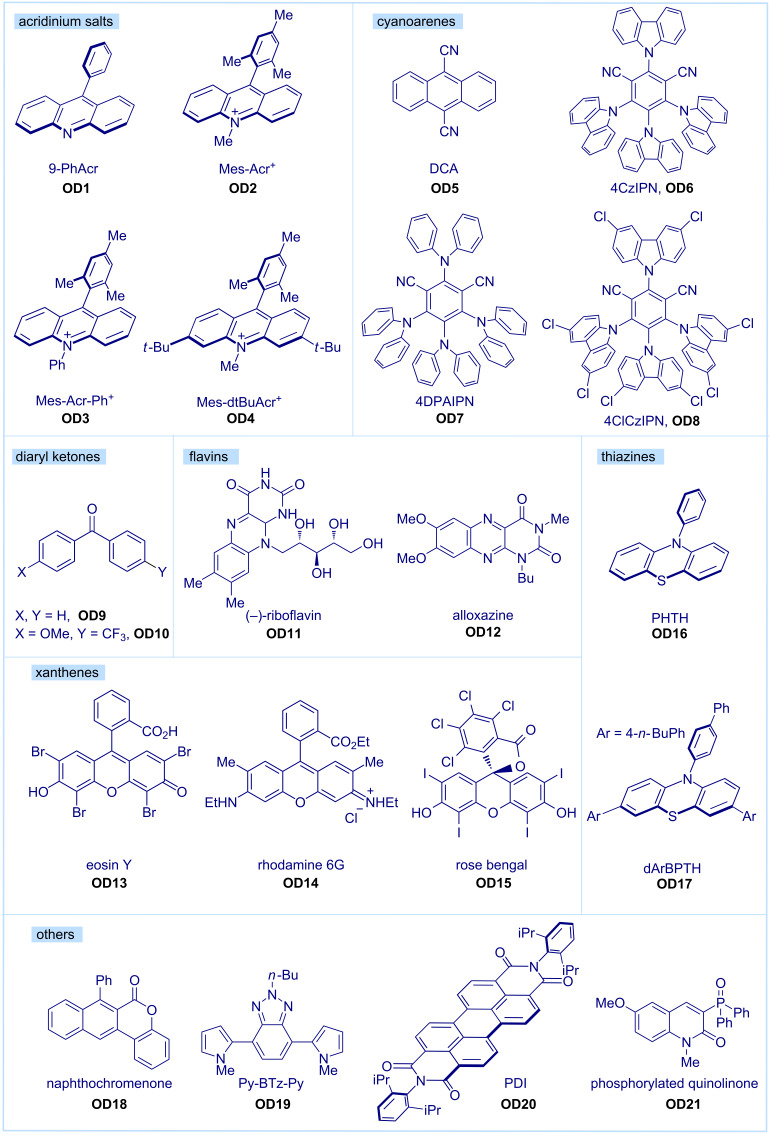
Selected examples of organic dyes. Mes-Acr^+^: 9-mesityl-10-methylacridinium, DCA: 9,10-dicyanoanthracene, 4CzIPN: 1,2,3,5-tetrakis(carbazol-9-yl)-4,6-dicyanobenzene, 4DPAIPN: 1,3-dicyano-2,4,5,6-tetrakis(*N*,*N*-diphenylamino)benzene, PHTH: 10-phenylphenothiazine, dArBPTH: 10-([1,1'-biphenyl]-4-yl)-3,7-bis(4-butylphenyl)-10*H*-phenothiazine, Py-BTz-Py: butyl-4,7-bis(1-methyl-1*H*-pyrrol-2-yl)-2*H*-benzo[*d*][1,2,3]triazole, PDI: *N*,*N*-bis(2,6-diisopropylphenyl)perylene-3,4,9,10-bis(dicarboximide).

In the last years, several classes of organic dyes, such as acridiniums (**OD1**–**4**), cyanoarenes (**OD5**–**8**), diaryl ketones (**OD9**/**10**), flavins (**OD11**/**12**), xanthenes (**OD13**–**15**), thiazines (**OD16**/**17**) and various other dyes, such as **OD18**–**21**, have been exploited (Figure 1), and the field of organic photocatalysis has been extensively covered by various reviews [16,19–25]. Most of these reports are organized according to the structural features of the dye and/or their applications in synthetic chemistry. In contrast, this review will focus on the different possible conceptual approaches based on organic photocatalysts for the generation of reactive intermediates. After a short introduction on activation modes in photocatalysis, selected case studies where organic dyes have been exploited for generating carbon-centered radicals, charged open-shell species, and heteroatom-centered radicals will be covered. The last short section will be devoted to activated organic substrates generated by energy transfer. C(sp) radicals will not be discussed. To the best of our knowledge, no report on an organophotocatalyzed generation of a C(sp) species has been disclosed yet. Each presented approach will be accompanied by one selected example, which we found particularly illustrative. This report is therefore in no means comprehensive, and readers searching to gain deeper insight into photocatalytic processes and/or for an exhaustive coverage of applications should refer to more complete specialized reviews and books [26–30]. We hope this report can motivate general synthetic chemists to consider photocatalytic approaches mediated by organic dyes as valuable tools for accessing important reactive intermediates and guide them in the first choice of a catalyst and a method.

### Activation modes in photocatalysis

Electronically excited photocatalysts interact with organic molecules via three main pathways: electron transfer (ET), EnT, and atom transfer (AT).

**Scheme 1:**
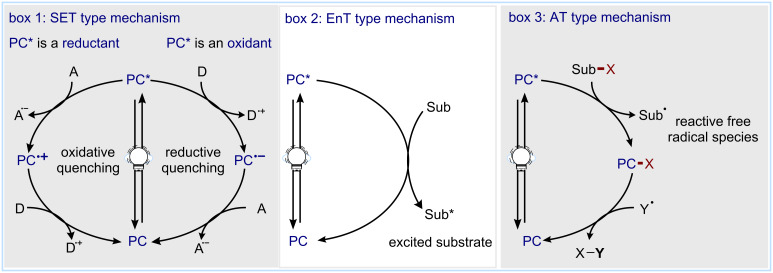
Activation modes in photocatalysis.

In the first case (Scheme 1, box 1), the excited photocatalyst (PC*) undergoes a single-electron transfer (SET) with a suitable electron acceptor **A** or electron donor **D**. In an oxidative quenching cycle, PC* acts as a reductant donating an electron to **A**. This generates the oxidized form of the photocatalyst, **PC^•+^**, and a reduced acceptor, **A^•−^**. Alternatively, in a reductive quenching cycle, PC* acts as an oxidant promoting an SET oxidation of the electron donor **D**. This leads to the reduced photocatalyst **PC^•−^** and the oxidized donor **D^•+^**. Following this initial SET, a second electron transfer must occur to ensure the catalyst turnover and restore the ground state photocatalyst: **PC^•+^** needs to be reduced by an electron donor **D**, whereas **PC^•−^** needs to undergo an oxidation by an electron acceptor **A**. In each of these steps, the role of **A** or **D** is assumed by a redox-active agent, either the substrate, a sacrificial electron donor/acceptor, or a reactive intermediate. This approach, usually named photoredox catalysis, has known a remarkable growth in the last decade and has given access to both neutral and charged radical species. The thermodynamic feasibility of these SETs is related to the redox potentials of the species involved. The values of the redox potentials discussed in this review are generally taken from the original publications described in the corresponding paragraphs.

In the second case (Scheme 1, box 2), the excited photocatalyst can engage in an EnT mechanism. Upon the excitation and intersystem crossing, the triplet state photocatalyst ^3^PC* can interact directly with a ground state species **Sub** and transfer the triplet energy to generate an excited state Sub* and the ground state **PC**. In most cases, Sub* is in the triplet state if it is an organic molecule. A common exception to this is molecular oxygen, which upon excitation attains a more reactive singlet state.

In the third mode of activation, atom transfer (AT, Scheme 1, box 3), the excited state photocatalyst PC* can abstract an atom, typically hydrogen, from a suitable substrate **Sub-X**, leading to the direct formation of an open-shell species, **Sub^•^**. In this case, the catalytic cycle is closed by a subsequent atom transfer that restores the ground state photocatalyst **PC**.

### C(sp^3^) radicals

Carbon-centered sp^3^ carbon (C(sp^3^)) radicals are important reactive intermediates for the construction of C–C and C–heteroatom bonds [31]. Their addition onto unsaturated systems, such as olefins and arenes, is particularly efficient. Additionally, alkyl radicals can undergo translocations, abstracting atoms from different sites. Recently, the ability of transition metal complexes to intercept alkyl radicals has been exploited for expanding the possibility of C–C bond formation reactions to cross-couplings. In all of these transformations, the substituents on the alkyl radical determine if it reacts as a nucleophile or an electrophile [32].

**Scheme 2:**
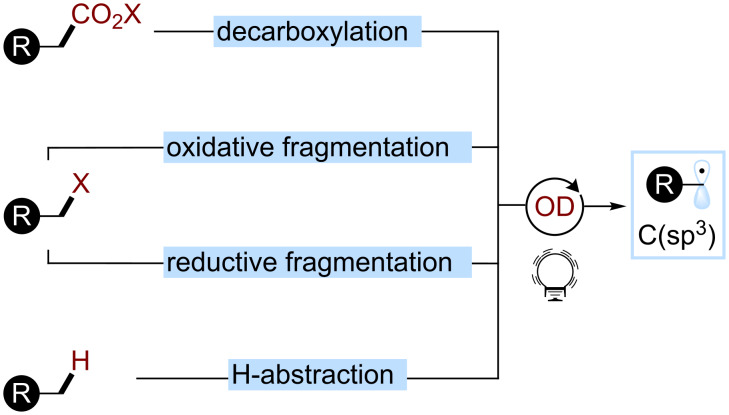
Main strategies for the formation of C(sp^3^) radicals used in organophotocatalysis.

Recently, photoredox catalysis has emerged as a powerful tool to access C(sp^3^) radicals, and organic dyes (ODs) have been demonstrated to act as competent photocatalysts for these light-mediated reactions. The main strategies used include decarboxylations from carboxylic acid derivatives (CO_2_X), such as carboxylates or RAEs (Redox Active Esters), oxidative fragmentations, and reductive fragmentations of various redox-active groups (X) as well as hydrogen atom abstractions (Scheme 2).

#### Decarboxylation

Carboxylic acids are naturally abundant functionalities that provide an easy access to C(sp^3^) radicals. Since the dawn of organic chemistry, several radical decarboxylations have been developed, including the Kolbe electrolysis [33–34], the Hunsdiecker reaction [35], and the Barton decarboxylation [36–38]. More recently, photoredox catalysis has appeared as a mild alternative to these venerable transformations [39–40], allowing the smooth generation of alkyl radicals from carboxylic acid derivatives. One of the main strategies for accessing C(sp^3^) radicals from carboxylic acids relies on the oxidation of the CO_2_H group. However, the high oxidation potential of these species makes them difficult to be directly activated by the excited state photocatalyst. For these reasons, the photocatalyzed decarboxylation often proceeds on the corresponding carboxylates, which are easier to be oxidized. This photoinduced SET, followed by the loss of CO_2_ as the sole byproduct, gives access to the desired C(sp^3^) radicals. Organic dyes are competent photocatalysts for these transformations, with many reports having appeared in the last five years.

**Scheme 3:**
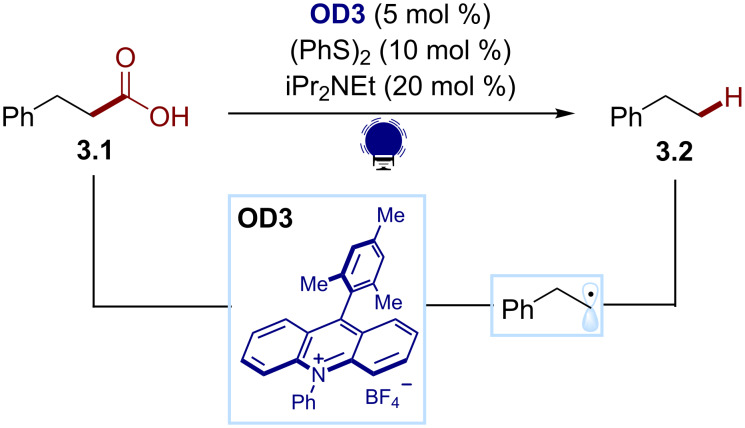
Illustrative example for the photocatalytic oxidative generation of radicals from carboxylic acids: the hydrodecarboxylation reported by Nicewicz and co-workers [41].

For example, Nicewicz developed a photocatalytic hydrodecarboxylation of primary, secondary, and tertiary carboxylic acids in 2015 (Scheme 3) [41]. The use of the strong oxidant Mes-Acr-Ph^+^ (**OD3**, *E(*PC^+^*/PC) ≈ 2 V) as organic photocatalyst leads to the oxidation/decarboxylation of the in situ-generated carboxylates (*E*_ox_ ≈ 1.3 V). An organic disulfide cocatalyst, (PhS)_2_, activated by the reduced photocatalyst, was found to act as a co-base (PhS^−^) and a hydrogen atom source (PhSH). The reaction allows the direct conversion of carboxylic acids, e.g., **3.1** to the corresponding alkanes, e.g., **3.2**. The scope includes carboxylic acids that were previously recalcitrant to other oxidative photocatalytic decarboxylations due to their high oxidation potential.

Other organic dyes, including several acridinium salts, have been successfully applied in organophotocatalytic decarboxylation protocols. For example, rhodamine 6G (**OD14**, *E(*PC^+^*/PC) ≈ 1.2 V) [42] was used for the photocatalytic decarboxylative azidation of cyclic amino acids and rose bengal (**OD15**) [43] for a decarboxylative amination of indoline-2-carboxylic acids with azodicarboxylate esters.

Another photocatalytic strategy for accessing C(sp^3^) radicals from carboxylic acids proceeds through a reductive decarboxylation pathway. This approach relies on the conversion of the acid into an easy reducible moiety, such as a redox-active ester (RAE). These species can accept one electron from the photocatalyst, and the ensuing reduced species releases the corresponding C(sp^3^) radical after a fragmentation and CO_2_ loss. This approach represents an alternative to oxidative decarboxylations, allowing the design of a photocatalytic cycle based on the SET reduction of the substrate. Furthermore, the relatively low reduction potential of these species (*E*_red_ ≈ −1.1 V for *N*-acetoxyphthalimide) [44] brings them into the operational range of various organic dyes, allowing mild reaction conditions.

**Scheme 4:**
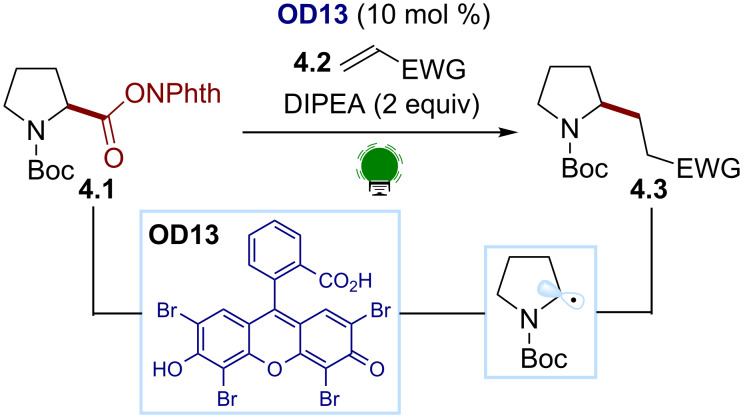
Illustrative example for the photocatalytic reductive generation of C(sp^3^) radicals from redoxactive esters: the decarboxylative Giese reaction reported by König and co-workers [45].

König reported a visible light-promoted photochemical reductive decarboxylation/alkylation of carboxylic acid analogs (Scheme 4) [45]. In this protocol, the carboxylic acids are converted into the corresponding RAE **4.1** by a condensation with *N*-hydroxyphthalimide. The organic dye eosin Y (**OD13**, *E(*PC/PC^−^) ≈ −1.1 V), in the presence of the sacrificial electron donor DIPEA, can reduce these species under green light irradiation. The ensuing decarboxylation provides a C(sp^3^) radical, which undergoes a radical conjugate addition with a suitable Michael acceptor **4.2**, providing the desired alkylation products **4.3**.

A similar strategy for radical generations was applied by Glorius and co-workers. They exploited a combination of organophotoredox and copper catalysis to achieve the conversion of carboxylic acids into alkenes using *N*,*N*-diaryldihydrophenazine as an organic photocatalyst [46]. Rose bengal (**OD15**) was also exploited as an organic photocatalyst to trigger the reductive fragmentation of phthalimide-based redox-active esters [47].

#### Other oxidative fragmentations

**Figure 2:**
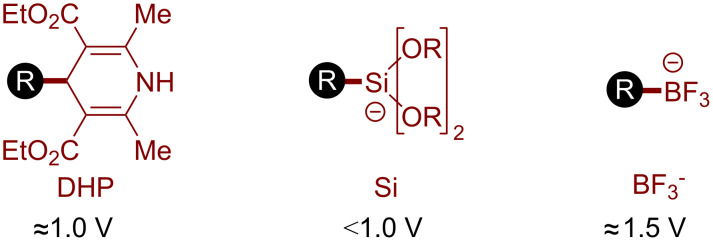
Common substrates for the photocatalytic oxidative generation of C(sp^3^) radicals.

In addition to the decarboxylation reactions, organic photoredox catalysis can be exploited to access C(sp^3^) radicals via the oxidative fragmentation of different redox-active functional groups (Figure 2) [48]. Among them, dihydropyridines (DHPs), silicates, and tetrafluoroborate salts were recently exploited in organophotocatalytic reactions. These functionalities can act as donors in reductive quenching manifolds and release the desired C(sp^3^) radicals after the fragmentation of the oxidized form. Due to their attitude towards SET oxidations, these substrates are valuable alkyl radical precursors; however, they are generally less available than carboxylic acids, and the fragmentations release stoichiometric amounts of byproducts.

**Scheme 5:**
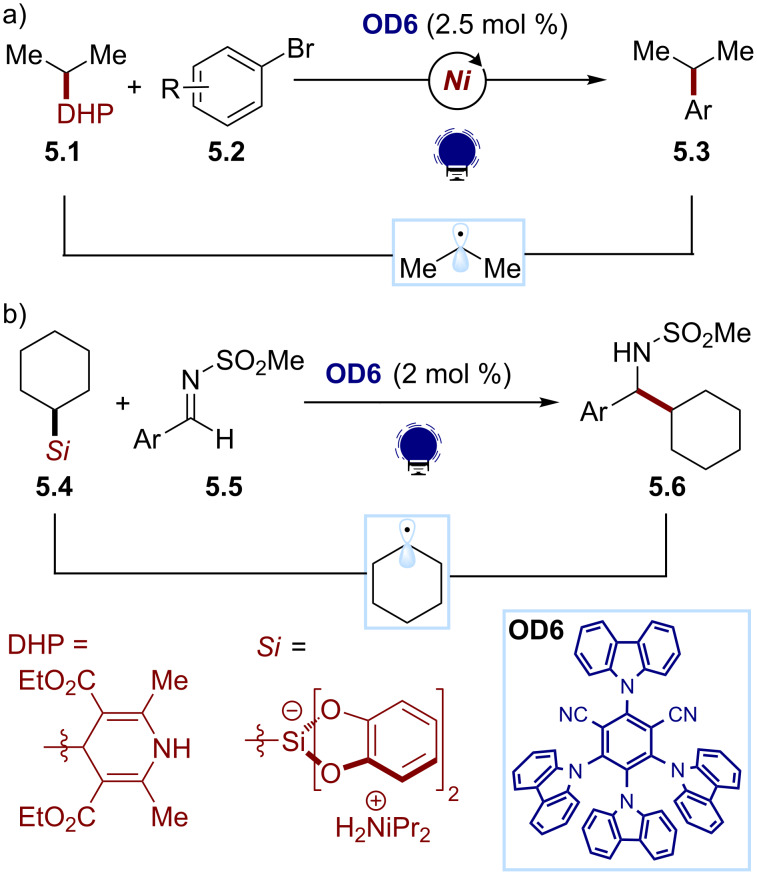
Illustrative example for the photocatalytic oxidative generation of radicals from dihydropyridines and silicates: the nickel-catalyzed cross-coupling with aryl bromides and addition to imines reported by Molander and co-workers [49–50].

The organic dye 4CzIPN (**OD6**), due to its oxidative abilities in the excited state (*E*(PC*/PC^−^) ≈ 1.35 V), has proved to be a versatile organic photocatalyst for accessing C(sp^3^) radicals through oxidative fragmentations. In particular, it has been used for generating an alkyl radical from the 4-alkyl-1,4-dihydropyridine (DHP) **5.1** in a metallaphotoredox protocol for the synthesis of alkylated (hetero)arenes (Scheme 5a) [49]. These substrates, easily synthesized from the corresponding aldehydes, can undergo a facile SET oxidation with the excited state of 4CzIPN. The ensuing fragmentation of the pyridyl group releases the C(sp^3^) radical, which is intercepted by an organometallic intermediate, obtained by the oxidative addition of a nickel catalyst to the (hetero)aryl bromide **5.2**. The desired (hetero)arene product **5.3** is obtained after the reductive elimination of the nickel complex. In this method, the reduced organic photocatalyst (*E*(PC/PC^−^) ≈ −1.2 V) is responsible for turning over the nickel catalytic cycle by reducing the Ni(I) intermediate.

Organosilicates can also be exploited as alkyl radical sources in organic photocatalytic reactions. For example, Molander and co-workers developed a photochemical protocol for the radical alkylation of imines using ammonium alkylbis(catecholato)silicates **5.4** as the C(sp^3^) radical precursors (Scheme 5b) [50]. 4CzIPN (**OD6**) was found again to be the best organic photocatalyst, triggering the oxidative fragmentation of the bis(catecholato)silicate. The so-formed alkyl radical adds to the imine substrate **5.5**, yielding the desired amine product **5.6**.

**Scheme 6:**
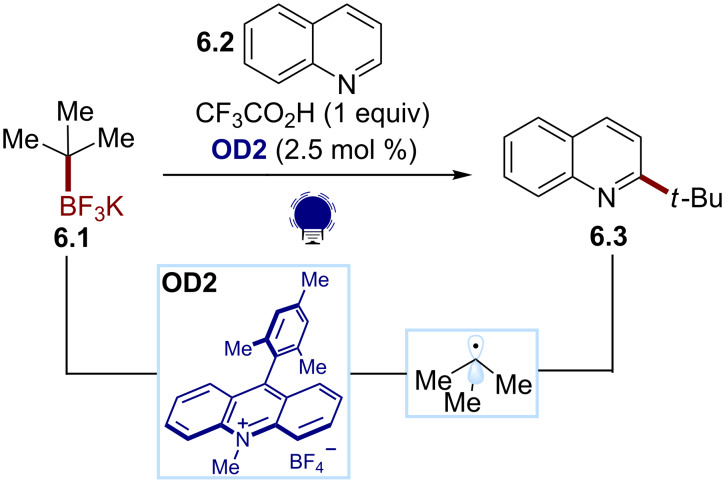
Illustrative example for the photocatalytic oxidative generation of C(sp^3^) radicals from trifluoroborates: the addition to quinolines reported by Molander and co-workers [51].

Finally, the same group developed a photochemical radical alkylation of heteroarenes with alkyl trifluoroborate salts (Scheme 6) [51]. In this reaction, the photoinduced oxidative fragmentation of the alkyl trifluoroborate salt **6.1** was promoted by the acridinium photocatalyst **OD2**. The resulting alkyl radical can be intercepted by a protonated heteroarene **6.2**. The addition of potassium persulfate leads to the regeneration of the photocatalyst and the rearomatization of the intermediate, delivering the desired alkylated heteroarene **6.3**.

#### Other reductive fragmentations

Another organophotocatalytic strategy for accessing C(sp^3^) radicals relies on the reductive homolytic cleavage of easily reducible functional groups. In this case, the substrates can act as acceptors in SET reductions, and the alkyl radical is obtained after the fragmentation of the reduced intermediates. Due to their availability and their attitude to undergo reductive fragmentations, alkyl halides have been extensively used as C(sp^3^) radicals sources.

**Scheme 7:**
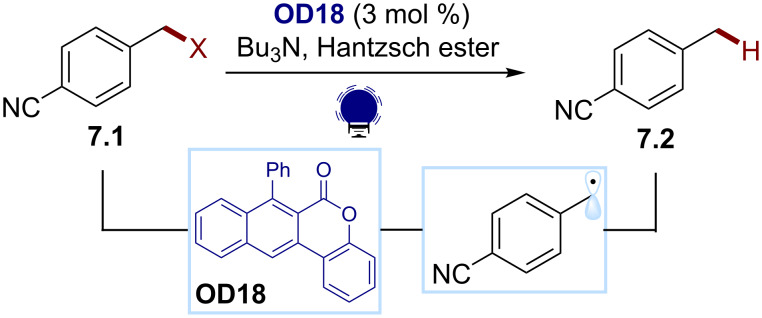
Illustrative example for the photocatalytic reductive generation of C(sp^3^) radicals from benzylic halides: the reduction with a Hantzsch ester reported by Dell’Amico and co-workers [52].

Recently, Dell’Amico and co-workers reported a new class of naphthochromenone-based organic dyes, which, due to the wide redox window (*E* = 3.22 eV, +1.65 V/−1.77 V), can be exploited as photocatalysts for various transformations, including the reductive dehalogenation of benzylic halides (Scheme 7) [52]. In this protocol, the excited state photocatalyst **OD18** can generate C(sp^3^) radicals through the reductive cleavage of various benzyl halides **7.1** (I, Br, Cl). The radical is then intercepted by an H atom donor (Hantzsch ester), which delivers the corresponding toluene derivative **7.2**.

Other organic dyes can promote the reductive fragmentation of alkyl halides. For instance, König and Zeitler demonstrated that a combination of eosin Y (**OD13**) with a sacrificial electron donor can trigger the reductive debromination of several α-carbonyl halides [53]. Riboflavin (**OD11**) [54] and thiaporphyrin [55] have been applied as well as organic photocatalysts for similar reductive dehalogenations.

#### Hydrogen atom transfer

**Scheme 8:**
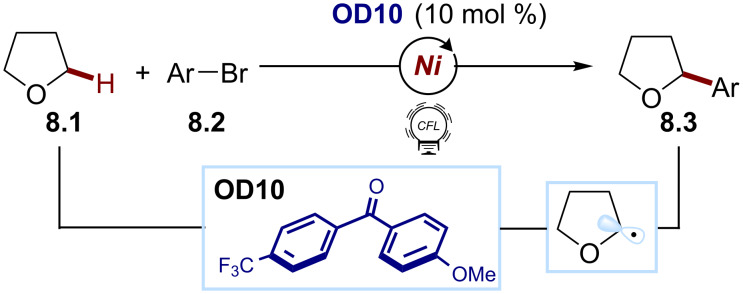
Illustrative example for the photocatalytic generation of C(sp^3^) radicals via direct HAT: the cross-coupling with aryl bromides reported by Martin and co-workers [57].

Photocatalytic hydrogen atom transfer (HAT) represents a valuable strategy for accessing C(sp^3^) radicals. This method allows the direct cleavage of a C–H bond and the consequent generation of alkyl radicals without relying on the presence of redox-active functional groups. This results in a superior atom economy compared to other methods for radical generation [56]. Within this field, organic dyes can act as competent photocatalysts for direct HAT processes. Specifically, upon light excitation, photoactive carbonyl compounds, such as benzophenone and its derivatives, reach an electronically excited triplet state: a biradical capable of abstracting an H atom from C–H bonds. Recently, Martin exploited this feature in a nickel-catalyzed process for the alkylation of arenes (Scheme 8) [57]. In this report, the excited state of a push–pull benzophenone **OD10** can abstract an H atom from the substrate tetrahydrofuran (**8.1**), giving access to the key C(sp^3^) radical. The nickel catalyst, after an oxidative addition with the aryl bromide **8.2** and intercepting the alkyl radical species, gave the radical cross-coupling product **8.3** upon a reductive elimination.

**Scheme 9:**
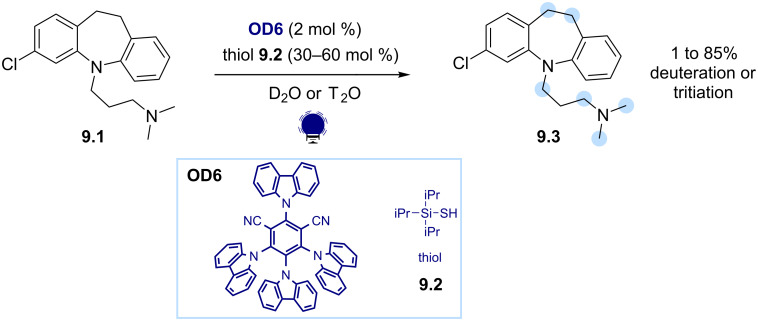
Illustrative example for the photocatalytic generation of C(sp^3^) radicals via indirect HAT: the deuteration and tritation of C–H bonds reported by MacMillan and co-workers [60].

Other diaryl ketone dyes, such as 9-fluorenone, have been successfully employed for the generation of C(sp^3^) radicals via HAT [58]. Interestingly, Wu and co-workers demonstrated that eosin Y (**OD13**) can also act as a direct HAT catalyst under visible-light irradiation [59]. Organic photoredox catalysis can also drive indirect HAT processes. In these reactions, the excited state of the photocatalyst engages in SET or energy transfer events with suitable cocatalysts for hydrogen shuttling, such as thiols. This results in the formation of active species that promote the H abstraction from the substrates. MacMillan exploited this strategy for the deuteration and tritiation of biologically relevant compounds using D_2_O and T_2_O as hydrogen isotope sources (Scheme 9) [60]. The SET oxidation of the thiol cocatalyst **9.2**, triggered by the excited photocatalyst 4CzIPN (**OD6**), generates a sulfur-centered radical capable of driving the HAT process with the substrate **9.1**. The trapping of the ensuing C(sp^3^) radical with the deuterated or tritiated thiol results in the incorporation of deuterium or tritium in multiple positions within the product **9.3**.

### C(sp^2^) radicals

#### Aryl radicals

**Scheme 10:**
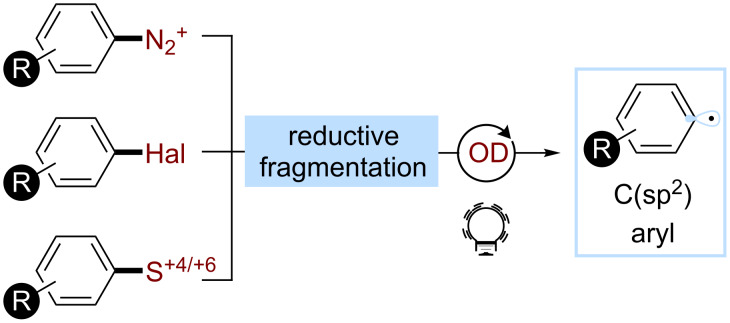
Selected precursors for the generation of aryl radicals using organophotocatalysis.

Considering the importance of arylation reactions in organic synthesis [61], aryl radicals are important intermediates to develop new synthetic methods [62]. Their reactivity was first explored in the Meerwein arylation as well as the Gomberg–Bachman and the Sandmeyer reaction [63–65]. Similar to C(sp^3^) radicals, aryl radicals add to unsaturated systems, such as (hetero)arenes, alkenes, or alkynes. They can also perform atom abstractions, in particular HATs and halogen abstractions [66]. The generation of such species generally occurs through the homolytic cleavage of a peripheral σ bond [67–68]. In the majority of the cases, a leaving group is reduced and fragments to the aryl radical. The precursors of choice encompass aryl diazonium salts, haloarenes, and sulfur(IV) or sulfur(VI) compounds (Scheme 10).

**Scheme 11:**
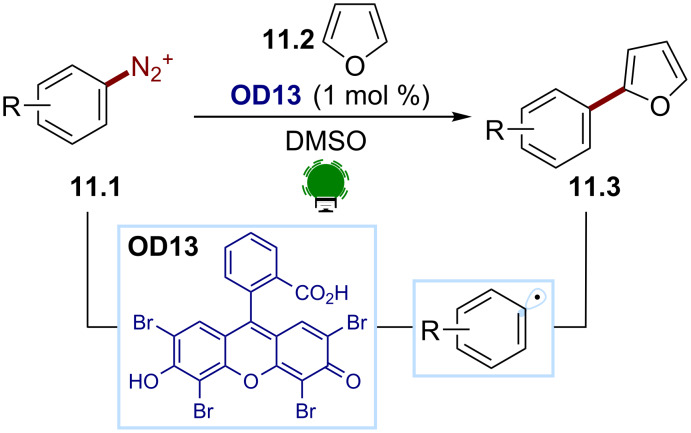
Illustrative example for the photocatalytic reductive generation of aryl radicals from aryl diazonium salts: the photocatalytic Meerwein arylation reported by König and co-workers [73].

**Aryl radicals from aryl diazonium salts.** Aryl diazonium salts are attractive substrates for accessing aryl radicals. Despite their intrinsic thermal instability and exothermic decomposition [69–70], these species are straightforward to synthesize and can be easily reduced (*E*_red_ ≈ −0.1 V to −0.3 V) [71–72]. This makes them valuable electron acceptors for photocatalytic strategies. The SET reduction results in the generation of an aryl radical upon the irreversible loss of N_2_ as the sole byproduct. As first reported by König and co-workers, organic dyes can be successfully employed as photocatalysts for accessing aryl radicals from these substrates [73]. In this work (Scheme 11), eosin Y (**OD13**) was used for the organophotocatalytic reduction of the aryl diazonium salts **11.1** under visible-light irradiation. The key aryl radical was trapped with heteroarenes, such as **11.2**, to give the arylation products **11.3**. Recently, similar methodologies for aryl radical generations have been developed exploiting flow techniques [74] and different organophotocatalysts, such as a metal-free porphyrin [75] and rhodamine 6G (**OD14**) [76].

**Aryl radicals from aryl halides.** Aryl halides are generally more difficult to reduce than aryl diazonium salts (*E*_red_ < −1.2 V) [77–78]. However, they are more available and bench-stable. Their reduction potential is dependent on the substitution pattern and on the nature of the halide: as a trend, aryl iodides are easier to reduce than aryl bromides and aryl chlorides [67,77]. Under organophotocatalytic conditions, the reduction can be achieved following two main strategies for accessing stronger reduction potentials: (a) tuning the electronics of the organic dye or b) tuning the stability of the reduced photocatalyst, allowing a second photoexcitation.

**Scheme 12:**
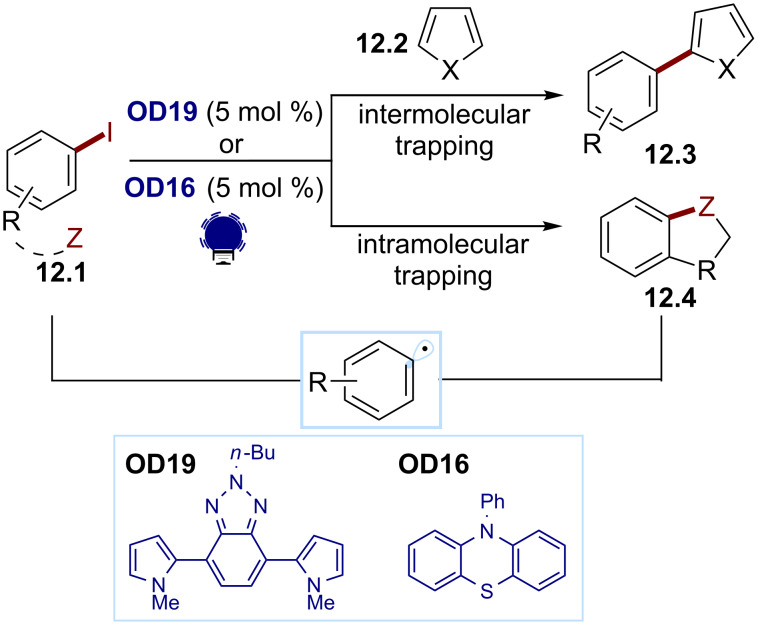
Illustrative examples for the photocatalytic reductive generation of aryl radicals from haloarenes: the photocatalytic heteroarylation of arenes reported by Zhang and co-workers (top) [79] and the intramolecular heterobicycle synthesis reported by Alemán and co-workers (bottom) [80].

For an example of the first strategy, Zhang and co-workers designed a new photocatalyst: Py-BTz-Py (**OD19**, Scheme 12), which was reducing enough (*E*(PC^•+^/PC*) ≈ −2.0 V) to activate the aryl iodides **12.1** for the synthesis of the arylated heteroarenes **12.3** via an intermolecular process [79]. Alemán and co-workers used PHTH (**OD16**) for the synthesis of various heteroatom-containing bicycles **12.4** through a tethered electrophile approach [80].

**Scheme 13:**
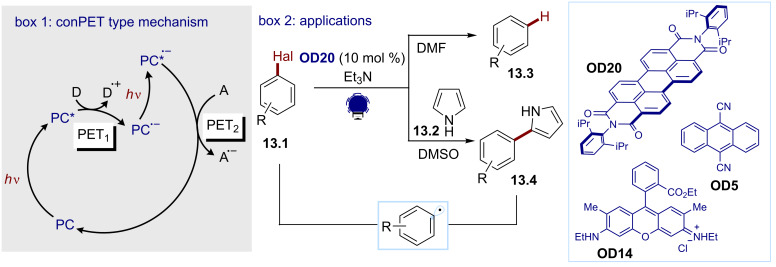
Illustrative example for the photocatalytic reductive generation of aryl radicals from aryl halides using a conPET strategy. Box 1: conPET mechanism and box 2: the dehalogenation and heteroarylation of aryl radicals reported by König and co-workers [81].

For the second strategy, König and co-workers developed an organic dye-based consecutive photoinduced electron transfer (conPET) strategy for the reduction of various aryl halides in 2014 [81]. As depicted in Scheme 13 (box 1), the conPET mechanism involves a first PET (PET_1_), where the excited photocatalyst (PC*) is reduced by a sacrificial electron donor **D**, such as a trialkylamine. The ensuing radical anion (**PC**^•^**^−^**), associated to an extended π-system, is persistent enough to absorb a second photon. The resulting excited state of the radical anion (**PC**^•^^−^*) is a strong reductant (*E*(PC/PC^•−^*) < −1.5 V), which can reduce the electron acceptor (**A**), resulting in a second PET (PET_2_). With this strategy, the authors could reduce a broad variety of aryl halides **13.1** using *N*,*N*-bis(2,6-diisopropylphenyl)perylene-3,4,9,10-bis(dicarboximide), (PDI, **OD20**) as a conPET photocatalyst for the generation of an aryl radical (box 2). The latter could be hydrogenated or heteroarylated to give the desired dehalogenated products **13.3** or arylheteroarenes **13.4**. Other organic dyes, such as dicyanoanthracene (**OD5**) and rhodamine 6G (**OD14**), have been successfully used in similar conPET strategies for the aryl radical-mediated derivatization of aryl bromides [82–83]. A similar double SET approach, where the first reduction of dicyanoanthracene (**OD5**) is achieved electrochemically, was recently disclosed by Lambert and Lin. In this report, the photoexcited radical anion of the dye was exploited for accessing aryl radicals as intermediates of a reductive borylation of aryl halides [84].

**Scheme 14:**
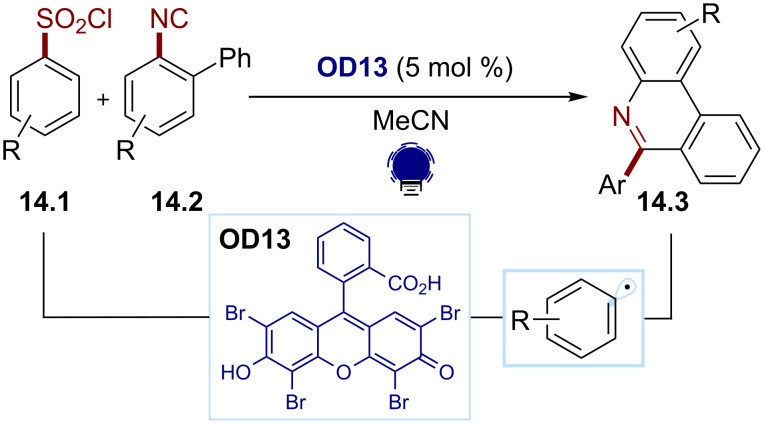
Illustrative example for the photocatalytic reductive generation of aryl radicals from arylsulfonyl chlorides: the phenanthridine synthesis via aryl radical addition to isonitriles reported by Gu and co-workers [85].

**Other sources of aryl radicals.** In addition to aryl diazonium salts and aryl halides, arylsulfonyl chlorides can be reduced with common organic dyes due to their relatively low reduction potential [78]. As an example, Gu and co-workers reported the use of eosin Y (**OD13**) for the SET reduction/desulfonylation of the arylsulfonyl chlorides **14.1** (Scheme 14) [85–86]. The ensuing aryl radical is trapped by an aryl isonitrile **14.2**, affording the bisarylation product **14.3**.

**Scheme 15:**
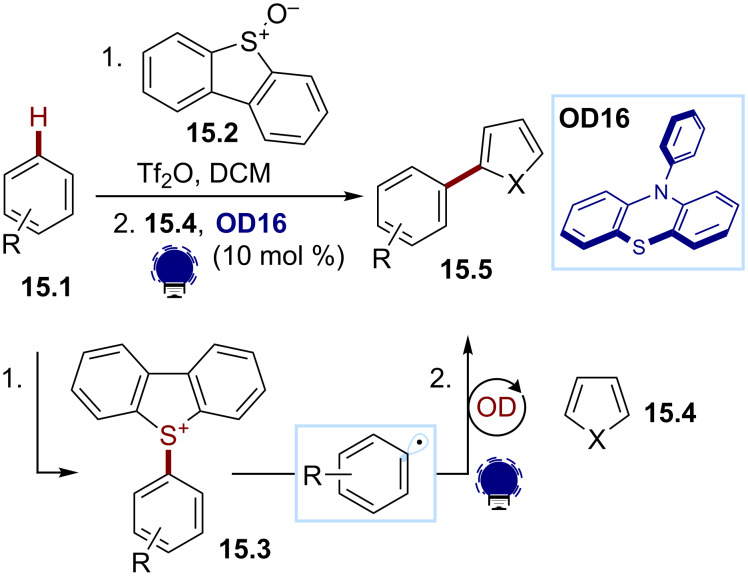
Illustrative example for the reductive photocatalytic generation of aryl radicals from triaryl sulfonium salts: the one-pot C–H heteroarylation of arenes reported by Procter and co-workers [87].

The direct organophotocatalytic activation of the C–H bond of an arene represents an atom-economical and attractive strategy for the generation of aryl radicals. Although inaccessible to date, a two-step strategy was recently developed by the Procter group. They reported a one-pot organophotocatalytic strategy for the heteroarylation of nonprefunctionalized arenes (Scheme 15) [87]. First, a triaryl sulfonium salt **15.3** is generated from the substrate **15.1** using dibenzothiophene *S*-oxide (DBTSO, **15.2**). Then, under visible-light irradiation, **15.3** could be reduced with PHTH (**OD16**) to generate an aryl radical that could further be trapped by a heteroarene **15.4**, such as pyrrole or thiophene, furnishing the arylation product **15.5**.

#### Acyl, oxylacyl, and carbamoyl radicals and their analogues

**Scheme 16:**
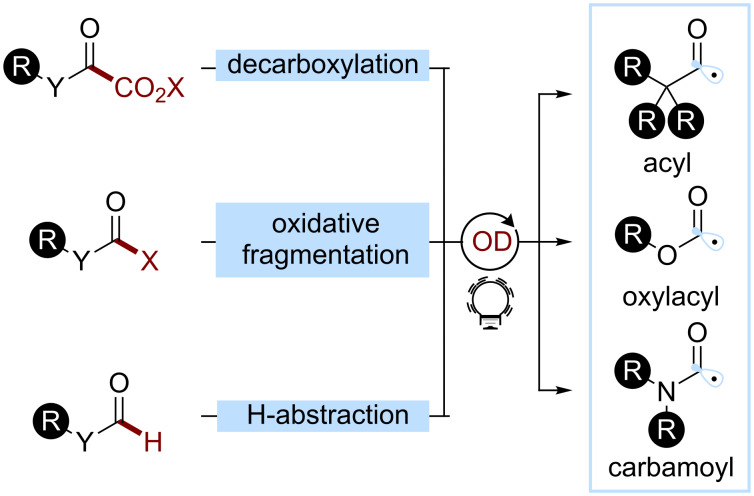
Main strategies towards acyl radicals used in organophotocatalysis.

The photocatalyzed generation of acyl radicals is of great interest as they are precursors for the synthesis of diverse carbonyl compounds [88]. The acyl radical is generally considered as a nucleophilic radical and reacts rapidly with electron-poor π-systems: heteroarenes and electron-poor alkenes in particular. Using the tools of organophotocatalysis, this reactive intermediate can be directly obtained from carbonyl derivatives, such as aldehydes, α-keto acids, oxalates, or carbamates through oxidative or reductive decarboxylations, oxidative fragmentation, or H-abstraction (Scheme 16).

**Scheme 17:**
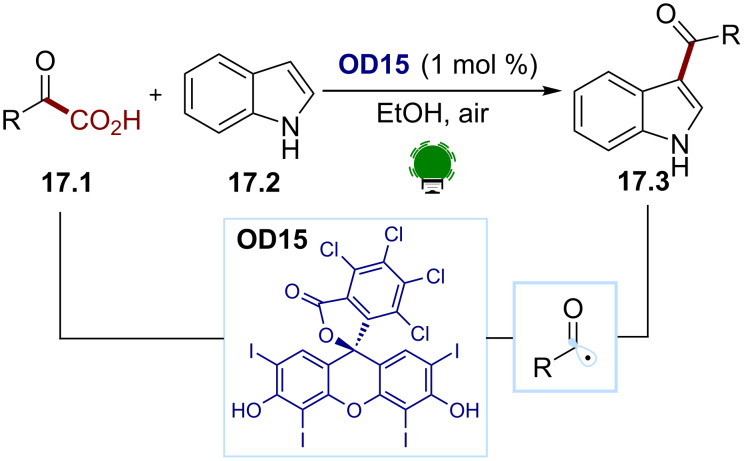
Illustrative example for the decarboxylative photocatalytic generation of acyl radicals from α-keto acids: the photocatalytic acylation of indole (**17.2**) reported by Wang and co-workers [89].

A common way to access such radicals is through the decarboxylation of α-keto acids. Both reductive and oxidative strategies were implemented. In 2016, Wang and co-workers developed an organophotocatalyzed acylation of indoles (Scheme 17) [89]. They successfully converted the aryl and alkyl α-keto acids **17.1** to the 3-acylindoles **17.3** using rose bengal (**OD15**) as a photocatalyst under aerobic conditions. Mechanistic studies suggest that rose bengal (**OD15**) acts as an energy transfer sensitizer generating singlet oxygen. The authors proposed that the latter would be responsible for the H atom abstraction from the α-keto acid. The α-keto carboxyl radical undergoes a decarboxylation, leading to the desired acyl radical.

**Scheme 18:**
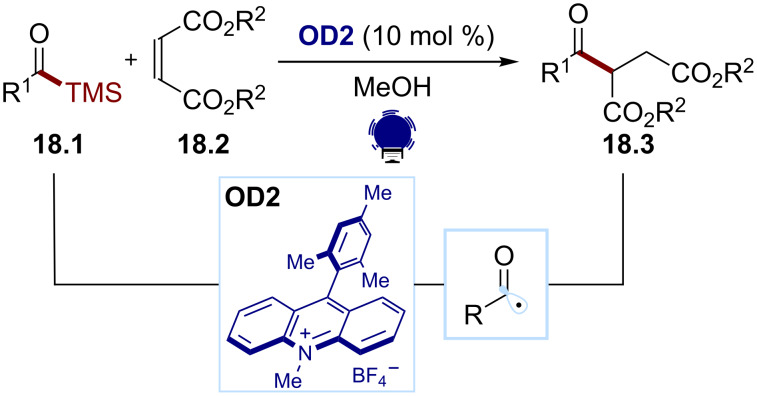
Illustrative example for the oxidative photocatalytic generation of acyl radicals from acyl silanes: the acylation of electron-poor olefins reported by Fagnoni and co-workers [90].

Acyl radicals can also be accessed through the oxidative cleavage of a redox-active group, such as acylsilanes or 1,4-dihydropyridine derivatives. In 2018, Fagnoni and co-workers developed an organophotocatalytic method to access nonsymmetrical ketones through the oxidation of the alkyl acylsilanes **18.1** (*E*_ox_ ≈ +1.3 V) by an acridinium photocatalyst **OD2** (*E*(PC^+^*/PC) ≈ 2.1 V, Scheme 18) [90]. After the fragmentation, the acyl radical can add to various Michael acceptors **18.2** to afford the desired ketone **18.3**.

**Scheme 19:**
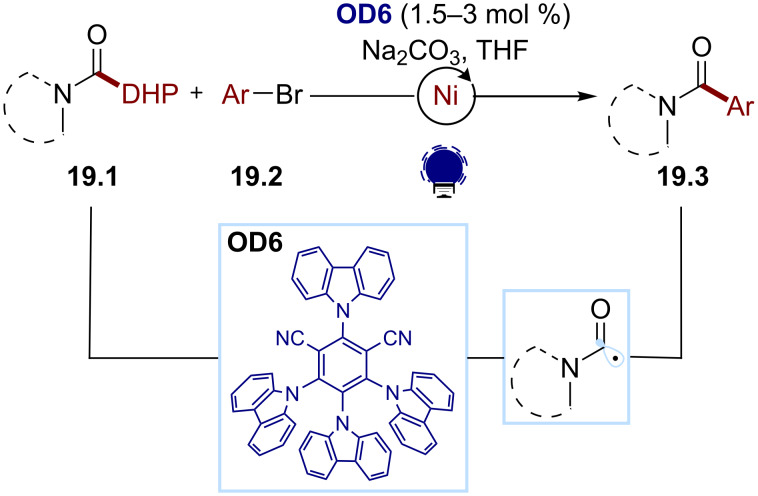
Illustrative example for the oxidative photocatalytic generation of carbamoyl radicals from 4-carbamoyl-1,4-dihydropyridines: the metallaphotoredox synthesis of aryl amides reported by Melchiorre and co-workers [91].

Recently, the Melchiorre group reported a carbamoyl radical-mediated metallaphotoredox synthesis of arylamides (Scheme 19) [91]. In this protocol, excited-state 4CzIPN (**OD6**) oxidizes a 4-carbamoyl-1,4-dihydropyridine **19.1**, which then fragments, releasing the corresponding pyridinium and the desired carbamoyl radical. The latter can be intercepted by an organonickel species resulting from the oxidative addition of the nickel catalyst to the aryl bromides **19.2**. The arylamides **19.3** are obtained following a reductive elimination, and the resulting Ni(I) species is reduced by the photocatalyst, and thus closing the catalytic cycle.

**Scheme 20:**
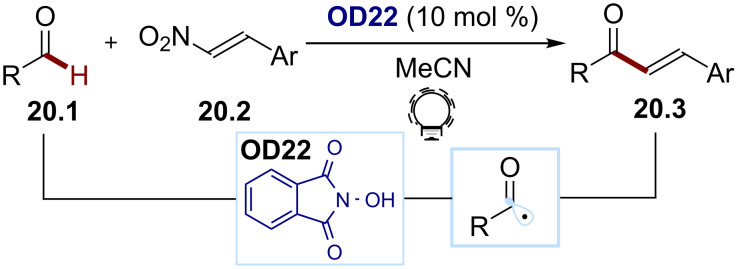
Illustrative example of the photocatalytic HAT approach for the generation of acyl radicals from aldehydes: the vinylation of aldehydes reported by Yadav and co-workers [92].

Finally, Yadav and co-workers developed a method for a chalcone synthesis using an H abstraction approach to access an acyl radical (Scheme 20) [92]. The authors proposed that the excited triplet state of the photocatalyst *N*-hydroxyphthalimide (NHPI, **OD22**, similar to the benzophenone photocatalysts **OD9** and **OD10**) can abstract an H atom from the aldehyde substrate **20.1**. The resulting acyl radical adds to the (*E*)-β-nitrostyrene **20.2**, and the following denitrosylation affords the chalcones **20.3**.

### Alkenyl and aryl radical ions (radical cations and radical anions)

**Scheme 21:**
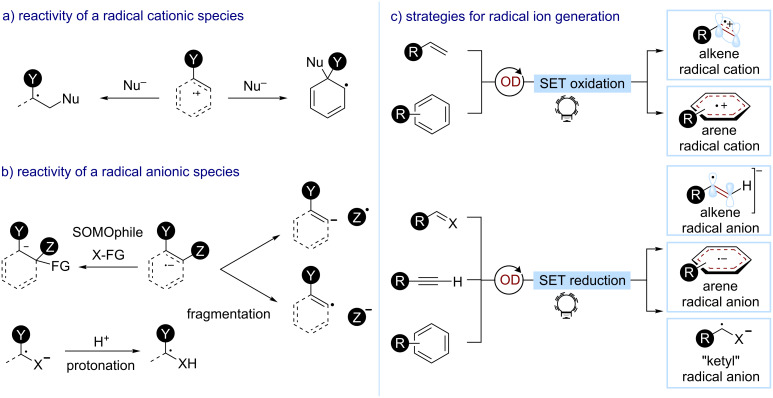
General reactivity of a) radical cations; b) radical anions; c) the main strategies towards aryl and alkenyl radical ions used in organophotocatalysis.

Recently, the exploitation of alkenyl and aryl radical ions has emerged as a platform for the functionalization of small molecules. They appear as attractive intermediates for a direct alkene difunctionalization or arene C–H functionalization. In particular, radical cations are strong electrophiles and undergo nucleophilic additions with two-electron donors (Scheme 21a) [93–94]. In contrast, radical anions can act as very strong single-electron nucleophiles and are often subject to fragmentation to give a neutral free radical and a charged species (Scheme 21b) [77,95]. When a fragmentation is not favored, these charged radicals can be intercepted and lead to different selectivities when compared to a traditional two-electron chemistry (e.g., anti-Markovnikov vs Markovnikov or *ipso*-S_N_Ar reactions). Heteroatom-containing radical anions, such as ketyl radicals, constitute a special class of substrates that usually undergo a protonation to form C(sp^3^) radicals.

Alkenyl or aryl radical ions are generally accessed through SET. The presence of electron-donating groups facilitates the oxidation of the precursor to the radical cations, while electron-poor alkynes and arenes can undergo SET reductions to generate the corresponding radical anions (Scheme 21c). Organophotocatalysis has been successfully exploited to promote these SETs and access charged alkenyl and aryl radicals. The reduction of carbon–heteroatom double bonds is especially easy and leads to the formation of ketyl radical anions or their equivalents with other heteroatoms. However, organophotocatalysts have been only rarely used for this type of reduction.

#### C_2_-charged radical species

**Scheme 22:**
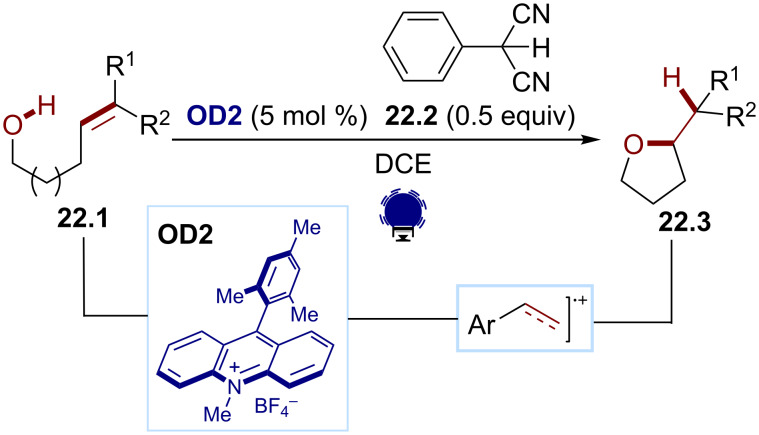
Illustrative example for the oxidative photocatalytic generation of alkene radical cations from alkenes: the hydroetherification of alkenes reported by Nicewicz and co-workers [98].

Since 2013, the Nicewicz group has developed a multitude of methodologies for the anti-Markovnikov hydrofunctionalization of alkenes, exploiting the reactivity of alkene radical cations generated using organic dyes [96–97]. Their seminal work reported the oxidation of the alkenols **22.1** by the Fukuzumi dye (**OD2**, Scheme 22) [98]. The so-formed radical cation undergoes an intramolecular nucleophilic 5/6/7-*exo*-*trig*-cyclization to give the cyclic ethers **22.3**. Mes-Acr-Me^+^ (**OD2**) is a strong enough oxidant (*E*(PC^+^*/PC) ≈ 2.1 V), allowing the oxidation of unactivated alkenes (1.2 ≤ *E*_ox_ ≤ 1.9 V). In this transformation, the cocatalyst **22.2** acts as an H atom shuttle. This alkene radical cation-based strategy has been extended to various hydrofunctionalizations of styrenes with mineral acids (hydrochloric acid, hydrofluoric acid, phosphoric acids, and sulfonic acids) [99]. During these studies, they optimized the structure of the Mes-Acr^+^ dye to improve the catalyst turnover. When changing the group on the nitrogen atom of the acridinium scaffold from an alkyl group (Me in **OD2**) to an aryl group (Ph in **OD3**), they could limit the photocatalyst bleaching due to the demethylation of the organic dye.

**Scheme 23:**
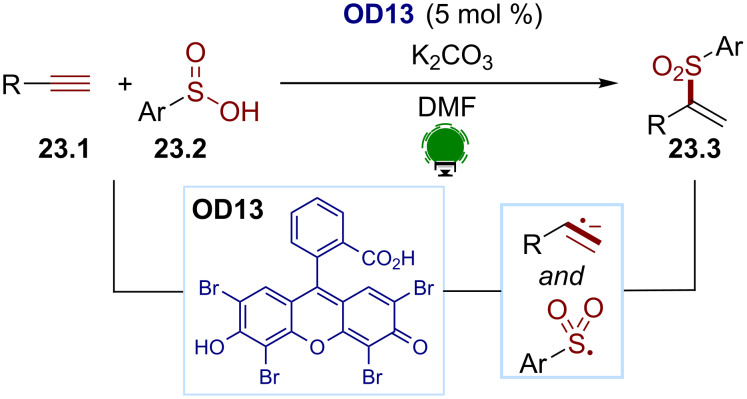
Illustrative example for the reductive photocatalytic generation of an alkene radical anion from alkynes: the Markovnikov-selective sulfonylation of alkynes reported by Lei and co-workers [100].

Alkene radical anions have been less exploited than their cationic equivalents. In 2017, the Lei group developed a Markovnikov-selective hydrosulfonylation reaction (Scheme 23) [100]. The alkynes **23.1** could be successfully converted to the vinyl sulfones **23.3** in the presence of the aryl sulfones **23.2** using eosin Y (**OD13**) as a photocatalyst. A tentative mechanism was proposed by the authors: under visible-light irradiation, the arylsulfinic acid could be oxidized to the corresponding sulfonyl radical by the excited state of eosin Y (**OD13**). The resulting reduced eosin Y^•−^ could then perform a reduction of the alkyne to generate a radical anionic species. The latter would be subsequently protonated before recombining with the sulfonyl radical to afford the desired vinyl sulfones.

#### C–X radical anions and derived neutral radicals

**Figure 3:**
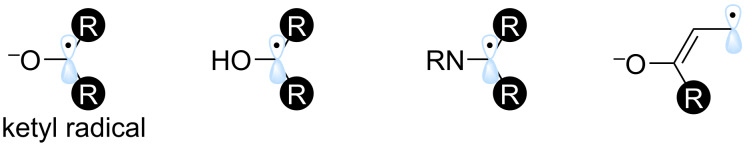
Structure of C–X radical anions and their neutral derivatives.

**Scheme 24:**
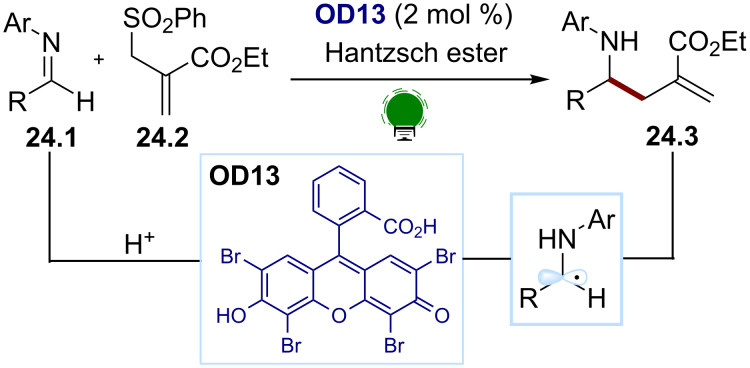
Illustrative example for the photocatalytic reduction of imines and the generation of an α-amino C(sp^3^) radical: the umpolung of imines for allylations reported by Dixon and co-workers [107].

Amidst charged radical species, ketyl radicals play a central role in organic synthesis (Figure 3). As intermediates, they are more stable because the charge is mainly localized on the oxygen atom. They are postulated to be the intermediate of important reactions, such as the pinacol [101] or the McMurry coupling [102]. Recently, photocatalysis has been used to access ketyl radicals through the reduction of ketones with a suitable transition metal-based photocatalyst [103] or organic dye [104]. The protonation of this type of radicals occurs on the heteroatom and leads to versatile neutral C(sp^3^) radicals. Such processes can also occur via concerted proton-coupled electron transfer mechanisms [105]. Similarly, the photocatalyzed reduction of imines followed by protonation, as well as the reduction of iminium compounds, gives access to α-amino radicals [106]. Most approaches are based on inorganic photocatalysts. As a rare example of the use of an organic dye, Dixon and co-workers used eosin Y (**OD13**) to reduce imines and generate the α-amino C(sp^3^) radical upon protonation (Scheme 24) [107]. This radical is then trapped with a suitable Michael acceptor **24.2** to obtain the corresponding allylation product **24.3** after a desulfonylation.

Enones can also be subject to SET reductions through photoredox catalysis, and this can lead to [2 + 2] cycloadditions [2,108]. Similar intermediates can also be generated through the direct activation of the ß-C–H bond [109]. However, organic dyes have not yet been broadly applied for such transformations.

#### Charged aryl radical species

Arene radical cations are valuable intermediates that enable the direct C–H functionalization of the aromatic species. They can be accessed through the oxidation of arenes under relatively strong oxidative conditions (*E*_ox_ > +1.0 V).

**Scheme 25:**
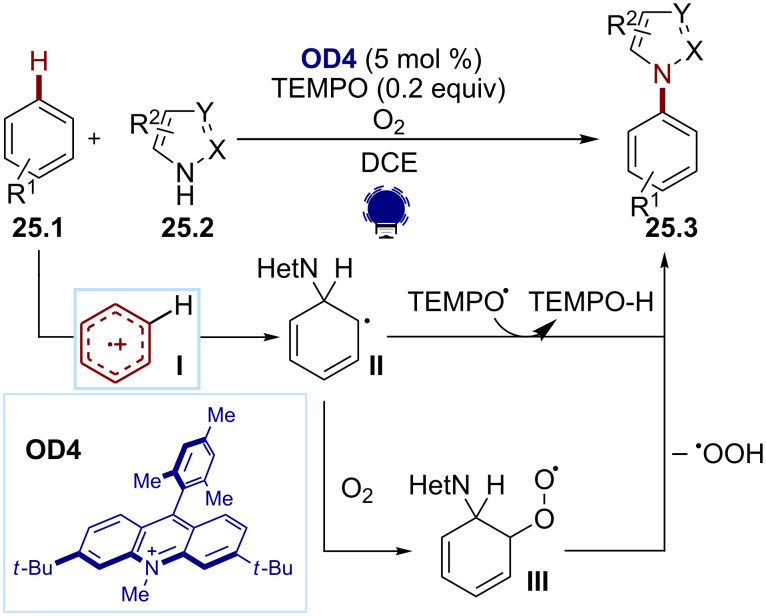
Illustrative example for the oxidative photocatalytic generation of aryl radical cations from arenes: the C–H amination of arenes reported by Nicewicz and co-workers [110].

Following up their work on alkene oxidations, Nicewicz and co-workers have developed several strategies for arene functionalizations through arene radical cation intermediates. Their work relies on the careful tuning of the Mes-Acr^+^ scaffold. In 2015, they developed a site-selective C–H amination of arenes (Scheme 25) [110]. The arenes **25.1** could be aminated by various N-heterocycles **25.2** for the synthesis of the C–H functionalization products **25.3** with fine-tuned Mes-di(*t*-Bu)Acr^+^ (**OD4**) as a photocatalyst. In this transformation, the arene is first oxidized by the excited state of the photocatalyst, generating the arene radical cation **I**. The latter then undergoes a nucleophilic attack of the N-heterocycle, affording the radical adduct **II**. TEMPO and O_2_ act as oxidants for the formation of the final aromatic compound via the peroxo radical **III** when using O_2_. Nicewicz and co-workers noticed that the presence of nucleophilic peroxide radicals generated from O_2_ led to the degradation of the classical Mes-Acr^+^ photocatalyst. The presence of bulky *tert*-butyl groups in the 3- and 6-positions provided a greater catalyst stability in presence of such nucleophilic radicals. This arene radical cation strategy has been further extended to cyanations, aminations, and fluorinations [111–116].

Arene radical anions, resulting from the reduction of aromatic compounds, are transient species that can enable aryl radical generations, in particular with haloarenes (see the section on aryl radicals from aryl halides). Notably, cyanobenzenes have been reported to undergo reductions and lead to *ipso*-substitution in place of the cyano group [117]. However, these methodologies rely on either a transition metal photocatalyst [118–119] or the formation of EDA complexes (electron donor–acceptor complexes) [120–123], and no method involving organophotocatalysts has been reported yet to the best of our knowledge.

### Nitrogen-centered radicals (NCRs)

Biologically relevant compounds often contain C–N bonds, rendering the development of efficient methodologies for their formation of paramount importance. Building on the seminal work of Zard [124], NCRs have emerged as key reactive intermediates. The reductive cleavage of weak N–X (X = halogen, S, O) bonds using a stoichiometric initiator or high temperatures set the path for key developments for the generations of NCRs, for instance, in the Hofmann–Löffler–Freytag reaction. With the rise of photoredox catalysis, they could be accessed under milder conditions in the last years [125–132].

**Scheme 26:**
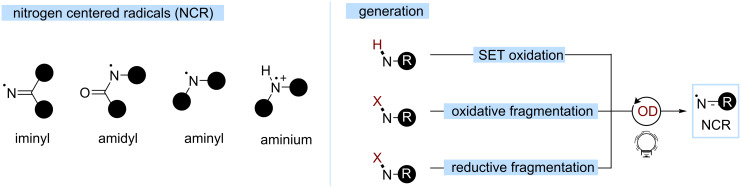
NCR classifications and generation.

NCRs can be organized into four main classes depending on the nitrogen atom hybridization and the substitution of the N atom, including iminyl, amidyl, aminyl, and aminium units (Scheme 26). The reactivity depends on the philicity of each species. In a general manner, NCRs are prone to the addition to unsaturated systems, either inter- or intramolecularly as well as HAT and Norrish type I fragmentations. Each of these steps generates a C-centered radical, later subject to termination. The variety of possible transformations has already been reviewed [125–132].

Iminyl radicals have an ambiphilic character. Amidyl radicals are electrophilic, with the substitution playing an important role [133–135]. As they are prone to efficient HAT, C(sp^3^)–H couplings at remote positions have been intensively studied [128,130,132,136]. Aminyl and aminium radicals display the opposite philicity: aminyls are nucleophilic, whereas upon protonation, the generated aminium is strongly electrophilic. N-Centred radical cations have been employed as key intermediates in amine syntheses [137–138]. These species can further react, according to the structure of the substrate, to form either iminium ions or C-centered radicals, such as α-amino radicals.

Several approaches for their efficient generation with organic dyes have been proposed, using SET oxidations and oxidative or reductive fragmentations. The hydroxylamine scaffold plays a prevalent role in the generation of various NCRs, both through oxidative or reductive fragmentation. *N*-aminopyridinium salts are also emerging starting materials.

#### Iminyl radical generation

Hydroxylamines have emerged as key precursors for iminyl radical generation [128]. Their redox properties can be easily tuned through the substitution pattern of the electrophore. They can be subjected to either oxidative or reductive SET with an excited photoredox catalyst. The most used scaffolds consist of electron-poor *O*-acyl and *O*-aryl hydroxylamines, which are prone to reduction, and α-*N*-oxy acids, which undergo oxidations followed by β-scission [128,136,139].

**Scheme 27:**
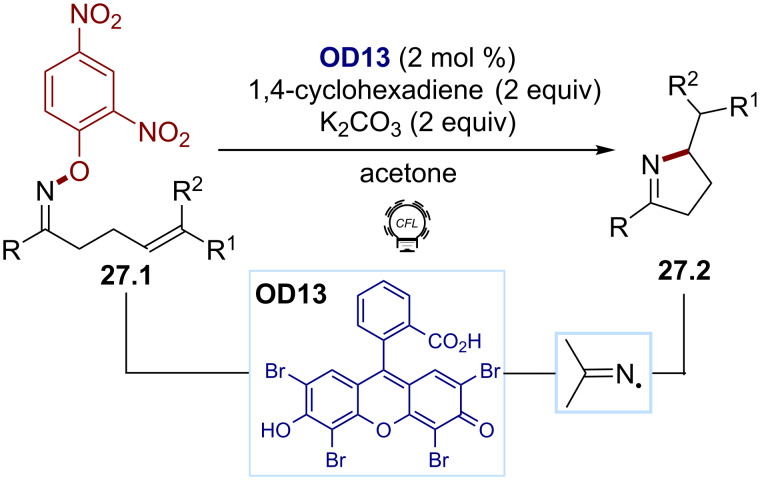
Illustrative example for the photocatalytic reductive generation of iminyl radicals from *O*-aryl oximes: the hydroimination/cyclization reported by Leonori and co-workers [140].

Leonori first described how *O*-aryl oximes **27.1** can be used to efficiently generate an iminyl radical in the presence of eosin Y (**OD13**, Scheme 27) [140]. This methodology was employed in hydroimination and iminohydroxylation cyclization reactions to give the pyrrolines **27.2**. The transformation proceeds via a SET reduction of the electron-poor aromatic moiety on the oxime to give a radical anion, which then fragments. Electrochemical studies highlighted that *O*-aryl hydroxylamines bearing nitro groups were within the reduction range of excited eosin Y (*E*_red_ between −0.55 and −0.93 V vs SCE, **OD13**, *E*(PC^•+^/PC*) ≈ −1.1 V).

**Scheme 28:**
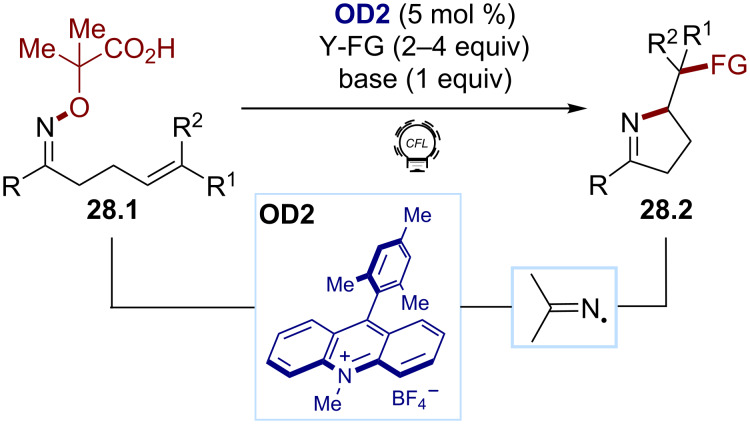
Illustrative example for the photocatalytic oxidative generation of iminyl radicals from α-*N*-oxy acids: the imino functionalization reported by Leonori and co-workers [141].

Oxidative strategies have also been described using the hydroxylamine scaffold, affording an efficient and general access to iminyl radicals. The first reports were released simultaneously by Leonori and Studer (Scheme 28) [141–142]. α-*N*-Oxy acid **28.1** carboxylates (*E*_ox_ = +1.6 V vs SCE for the caesium carboxylate) can be oxidized by the strongly oxidizing dye Mes-Acr-Me^+^ (**OD2**, *E*(PC^+^*/PC) ≈ 2.1 V). After the decarboxylation and β-scission, the corresponding iminyl radicals were submitted to various radical traps, Y-FG, to provide diverse imino-functionalized products **28.2**. A large variety of functionalizations was possible, such as halogenations, azidations, olefinations, or alkynylations. This activation mode was employed by the same group in a cascade strategy, which allowed a remote functionalization [143]. To extend the scope of such transformations, our group reported novel organic dyes in 2018, such as 4ClCzIPN (**OD8**), obtained through fine-tuning of 4CzIPN (**OD6**), which are both strong oxidants and reductants [144].

**Scheme 29:**
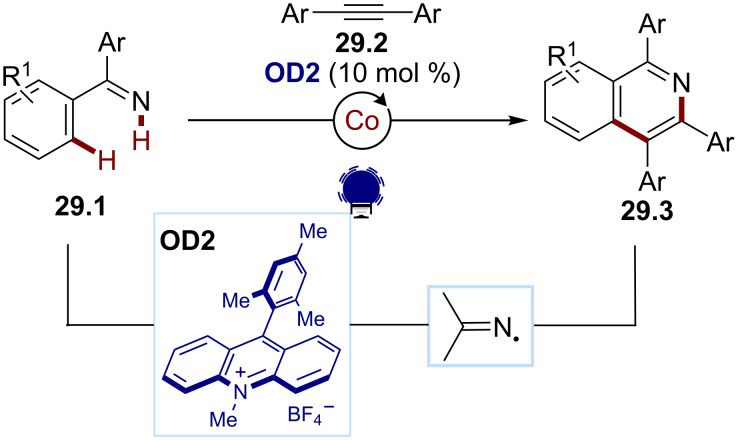
Illustrative example for the photocatalytic oxidative generation of iminyl radicals via an N–H bond oxidation: the radical cyclization reported by Li and co-workers [145].

In the search for a more atom-economical approach, the group of Li developed an iminyl radical formation by the N−H bond cleavage from the diarylimines **29.1** (Scheme 29) [145]. The direct oxidation of **29.1** was achieved using Mes-Acr-Me^+^ (**OD2**) and a cobalt cocatalyst, ensuring an efficient dehydrogenation. Various N-heterocycles **29.3** were accessed via a radical cyclization cascade with the alkyne derivatives **29.2**.

#### Amidyl radical generation

The oxidation of amides is more difficult compared to amines, with reduction potentials from 1.2 to 2.0 V vs SCE [138]. This has rendered the development of a general strategy for direct amidyl radical formations challenging. A successful approach has been the incorporation of a photolabile moiety on the nitrogen atom. Similar to the iminyl radical generation, the use of hydroxylamine derivatives has turned out to be very efficient [128,139].

**Scheme 30:**
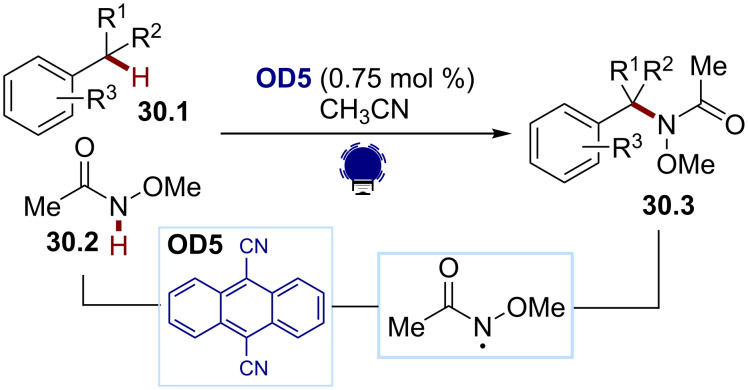
Illustrative example for the photocatalytic oxidative generation of amidyl radicals from Weinreb amides: the cross-dehydrogenative benzylic C(sp^3^)–H amination reported by Pandey and Laha [146].

The generation of amidyl radicals using organophotoredox catalysis was first reported by Pandey and Laha in 2015 (Scheme 30) [146]. They developed an intermolecular cross-dehydrogenative benzylic C(sp^3^) amination between the aryl substrates **30.1** and the amides **30.2** for the synthesis of the Weinreb amides **30.3** using DCA (**OD5**) as an organic dye. Under visible-light irradiation, the SET oxidation of **30.2** by the excited state of DCA, followed by a deprotonation, afforded the amidyl radical. This radical behaved as a powerful HAT reagent, allowing the formation of the benzylic radical from **30.1**. The absence of any activating group on the nitrogen renders this process atom-economical.

**Scheme 31:**
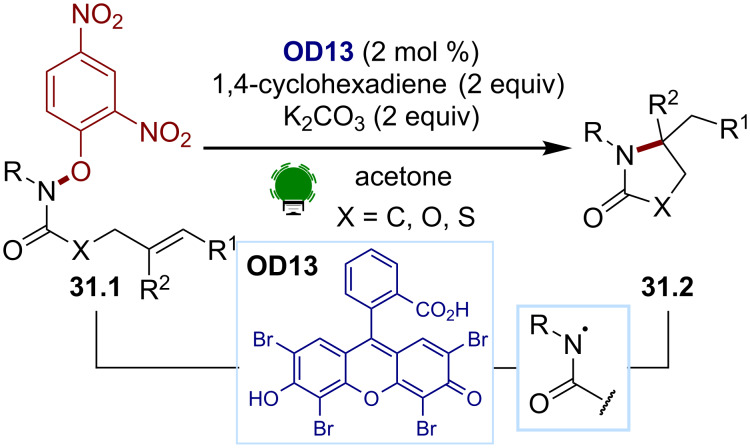
Illustrative example for the photocatalytic reductive generation of amidyl radicals from hydroxylamines: the hydroamination/cyclization reported by Leonori and co-workers [134].

In 2016, the Leonori group paved the way for the generation of amidyl radicals by SET reductions of hydroxylamines (Scheme 31) [134]. The previously described approach for iminyl radical formations was employed [140]. The same tunable aryloximes **31.1** were used as electrophores to generate the desired amidyl radicals. This methodology allowed the development of a hydroamination/cyclization cascade to give the heterocycles **31.2** and intermolecular *N*-arylations. A modified electrophore, *p*-CF_3_-substituted hydroxamide, was later proposed by the Yu group with a different dye, (2,4,6-tri(9*H*-carbazol-9-yl)-5-chloroisophthalonitrile (3CzClIPN) [147].

**Scheme 32:**
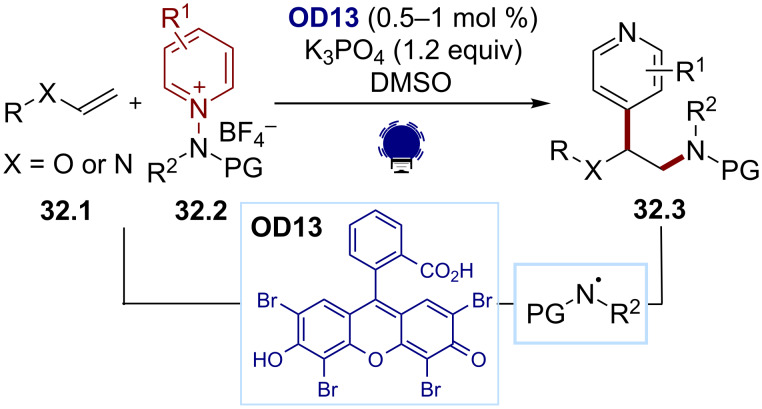
Illustrative example for the photocatalytic reductive generation of amidyl radicals from *N*-aminopyridinium salts: the intermolecular alkene 1,2-aminopyridylation reported by Hong and co-workers [148].

The *N*-aminopyridinium salts **32.2** are another scaffold for the generation of amidyl radicals via SET. After the reduction, a fragmentation affords the desired amidyl radicals together with the corresponding pyridines. The reduction potential of such species was around −0.75 V vs Ag/Ag^+^, depending on the substitution pattern. This scaffold was employed recently by the Hong group to achieve an intermolecular alkene 1,2-aminopyridylation to give the difunctionalized products **32.3** (Scheme 32) [148]. Eosin Y (**OD13**) was an efficient dye for the SET reduction of the *N*-aminopyridinium salts, which act as bifunctional reagents as the released pyridine later added to the olefin **32.1**.

**Scheme 33:**
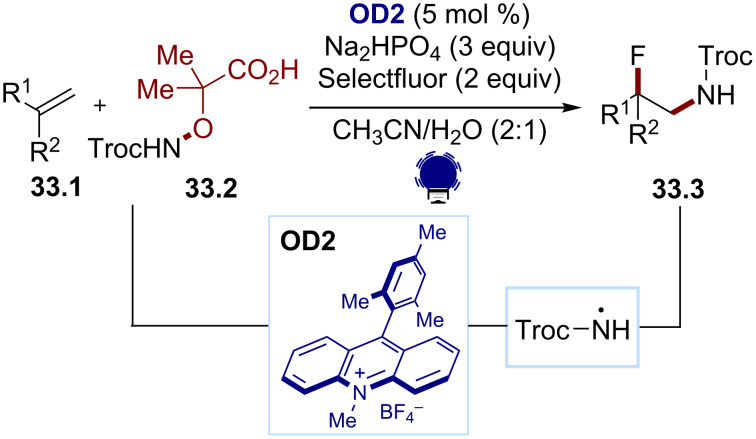
Illustrative example for the photocatalytic oxidative generation of amidyl radicals from α-amido-oxy acids: the amidofluorination of unactivated alkenes reported by Studer and co-workers [149].

The generation of amidyl radicals by the SET oxidation of hydroxylamines has also been explored. As for iminyl radicals, this enables the trapping of the subsequently formed carbon-centered radicals with various reagents. Studer and co-workers disclosed an amidofluorination of unactivated alkenes and the styrenes **33.1** to give the fluorinated amides **33.3**, employing the α-amido-oxy acids **33.2** and Mes-Acr-Me^+^ (**OD2**, Scheme 33) [149]. The same electrophore allowed a remote functionalization of amides to take place, as disclosed by the Leonori group [135]. 4CzIPN (**OD6**) was reported to be a suitable dye for the SET oxidation of such carboxylates.

#### Aminyl and aminium radical generation

The prevalent strategy for accessing aminium radicals is SET oxidations. The efficiency of this approach is closely related to the stabilization of the generated radical on the substrate (delocalization, electronic effects) or through a strain release ring-opening driving force.

**Scheme 34:**
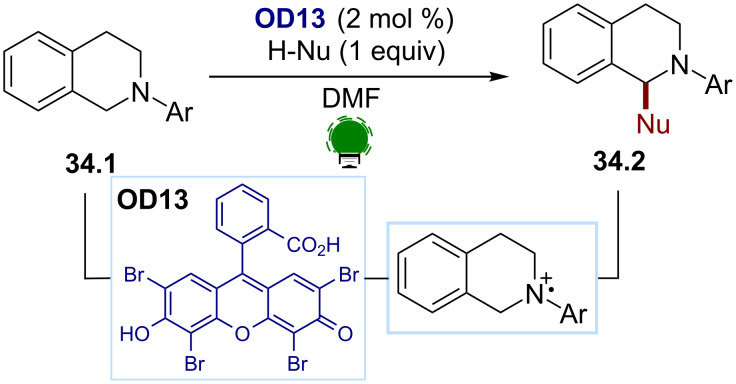
Illustrative example for the photocatalytic oxidative generation of aminium radicals: the *N*-aryltetrahydroisoquinoline functionalization reported by König and co-workers [150].

*N*-Aryltetrahydroquinolines **34.1** have been intensively studied as a privileged scaffold for N-centred radical cation formations [138]. After iminium formation, formed by subsequent HAT on the amine radical cation, various nucleophiles can add, resulting in diverse functionalizations. In 2011, König and co-workers [150] demonstrated that eosin Y (**OD13**) was an efficient dye for an aza-Henry reaction [151], affording the tetrahydroquinolines **34.2** (Scheme 34). Simultaneously, the Tan group described the use of rose bengal (**OD15**) in the same transformation [152].

**Scheme 35:**
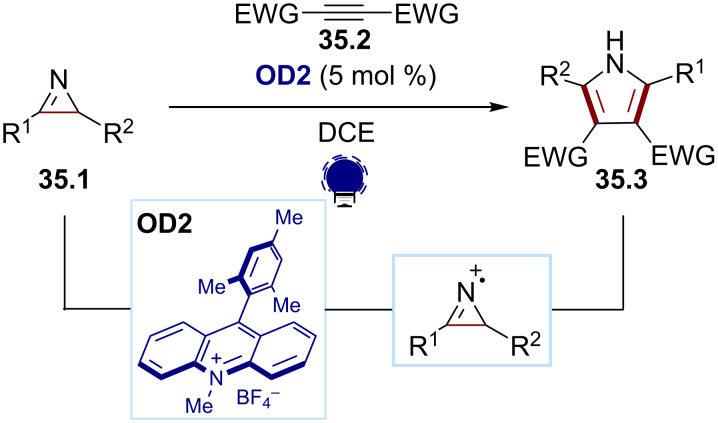
Illustrative example for the photocatalytic oxidative generation of nitrogen-centered radical cations from 2*H*-azirines: the pyrrole synthesis reported by Xiao and co-workers [153].

Regarding the strain release approach, the Xiao group reported a synthesis of the pyrroles **35.3** in 2014 [153]. They can be accessed via a photocatalytic formal [3 + 2] cycloaddition of the 2*H*-azirines **35.1** and the alkynes **35.2** (Scheme 35). The use of Mes-Acr-Me^+^ (**OD2**) was successful due to its high reduction potential in the excited state, together with an intense absorption in the visible-light region. The transformation proceeds via the single-electron oxidation of 2*H*-azirines, forming a nitrogen-centered radical cation, which is prone to ring opening.

**Scheme 36:**
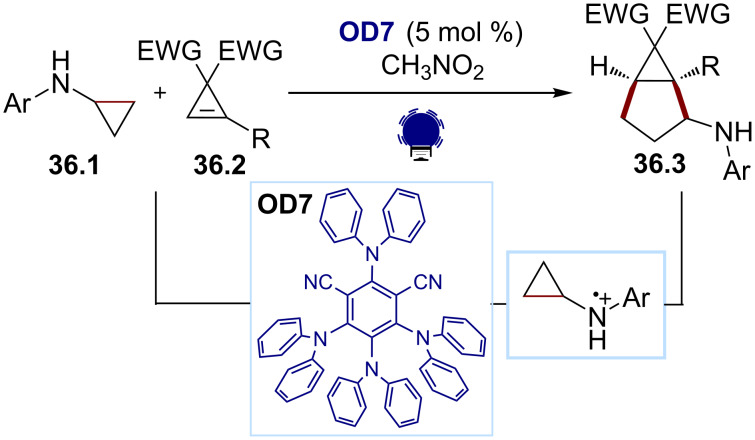
Illustrative example for the photocatalytic oxidative generation of nitrogen-centered radical cations from cyclopropylanilines: the [3 + 2] annulation of cyclopropylanilines and cyclopropenes reported by our group [154].

The driving force of the ring strain release and irreversible ring opening of nitrogen-containing small rings was also exploited by our group in 2019 (Scheme 36) [154]. Starting from the aminocyclopropanes **36.1** and the cyclopropenes **36.2**, a [3 + 2] annulation led to the bicyclo[3.1.0]hexanes **36.3**. Under visible-light irradiation, the excited-state photocatalyst **OD7** performs an SET oxidation of an cyclopropylaniline **36.1**, leading to an N-centred radical cation after ring opening. The latter undergoes a [3 + 2] annulation with cyclopropenes **36.2**, affording a bicyclic product **36.3**. The key to the broad substrate tolerance relied on using 4DPAIPN (**OD7**), which is a mild oxidant but a strong reductant (*E*(PC*/PC^•−^) = +0.90 and *E*(PC/PC^•−^) = −1.65 V vs SCE) [155].

**Scheme 37:**
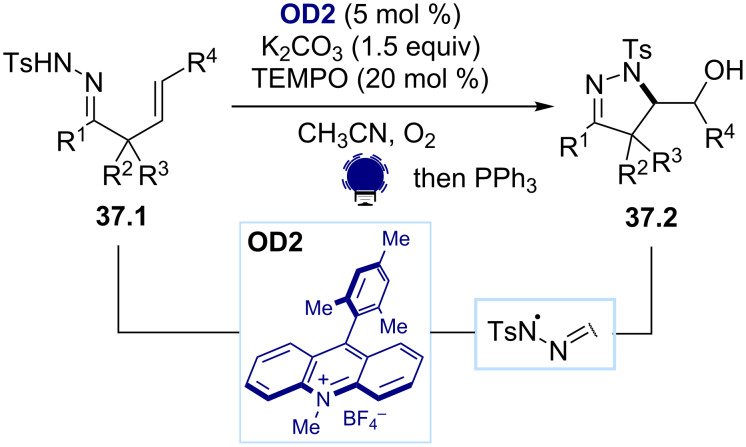
Illustrative example for the photocatalytic oxidative generation of hydrazonyl radical from hydrazones: the pyrazoline synthesis reported by Xiao and co-workers [156].

As another class of nitrogen radicals, hydrazonyl radicals can be formed from the direct N–H bond oxidation of the hydrazones **37.1**, as reported by the Xiao group (Scheme 37) [156]. This strategy was based on a cooperative TEMPO and photoredox catalysis. The SET oxidation of the anion of the β,γ-unsaturated hydrazones **37.1** is mediated by TEMPO^+^, itself formed by an SET oxidation with the excited state of Mes-Acr-Me^+^ (**OD2**). The pyrazolines **37.2** are formed through an intramolecular addition to the alkene, followed by a reaction with oxygen.

Finally, a remote functionalization of protected amines was also disclosed by Leonori and co-workers in their report on amidyl radical formations by SET oxidations of α-oxyamido acids using 4CzIPN (**OD6**, Scheme 28) [135].

### Oxygen-centered radicals

**Scheme 38:**
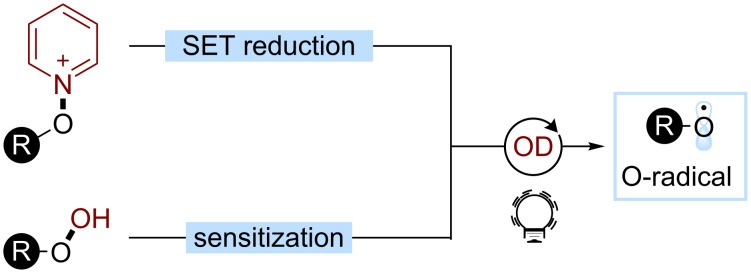
Generation of O-radicals.

Oxygen-centered radicals (O-radicals) represent an important class of heteroatom-centered radicals. In particular, their ability to promote radical translocations, especially HATs, and to undergo β-fragmentations makes them valuable reactive intermediates in organic synthesis [157–161]. Organic photoredox catalysis can be exploited to access O-radicals from different classes of substrates. In particular, *N*-alkoxypyridinium and alkyl hydroperoxides have been exploited as competent O-radical sources (Scheme 38). These substrates can be activated through SET reduction and energy transfer, respectively. The cleavage of the N–O or O–O bond releases the desired O-radical.

**Scheme 39:**
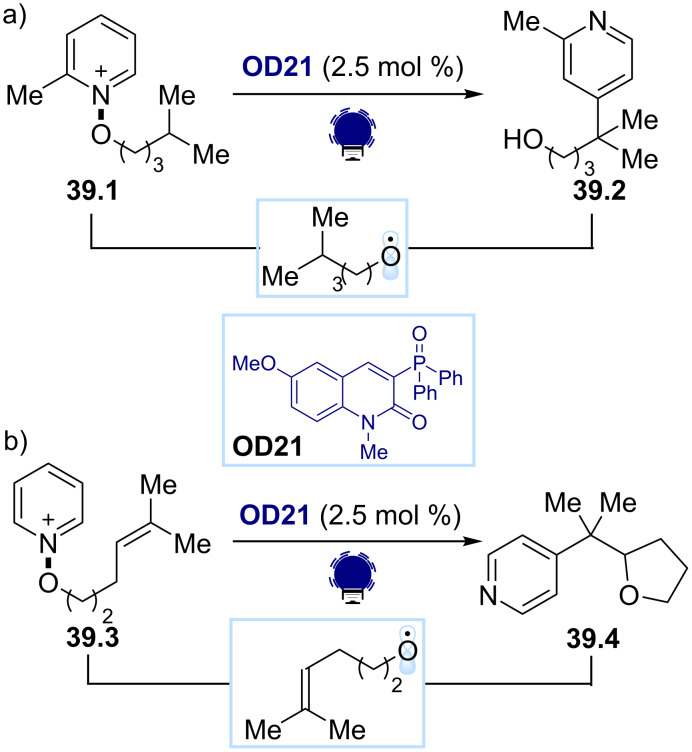
Illustrative examples for the photocatalytic generation of O-radicals from *N*-alkoxypyridinium salts reported by Hong, Baik and co-workers [160–161].

The ability of *N*-alkoxypyridinium salts to generate O-radicals under organophotocatalytic conditions was exploited by Hong, Baik, and co-workers (Scheme 39a) [160]. They reported the use of the phosphorylated quinolinone derivative **OD21** as a photocatalyst, which triggers the photoinduced SET reduction of the *N*-alkoxypyridinium salt **39.1**, leading to the formation of the key O-radical. This species rapidly undergoes a 1,5-HAT. The formed nucleophilic C-centered radical then adds selectively onto the C4 position of another pyridinium substrate **39.1**. The formed *N*-alkoxylated intermediate is converted into the desired product **39.2** after oxidation and releases another alkoxy radical at the same time. The measured quantum yield (Φ = 4.4) indicates that a chain mechanism is operative. Recently, the same group applied a similar organophotocatalytic system to perform a ring closure/pyridylation radical cascade for the synthesis of tetrahydrofuran derivatives (Scheme 39b) [161].

Eosin Y (**OD13**) has been demonstrated to be another competent photocatalyst for the generation of O-centered radicals from *N*-alkoxypyridynium salts. These radicals have been used as initators for the synthesis of highly functionalized benzo[*b*]phosphole oxides from arylphosphine oxide and alkynes [162].

**Scheme 40:**
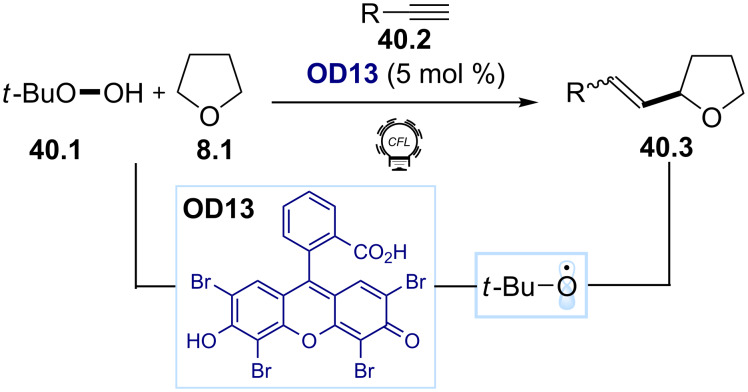
Illustrative examples for the photocatalytic generation of O-radicals from alkyl hydroperoxides: the vinylation of tetrahydrofurans reported by Wang and co-workers [165].

Alkyl hydroperoxides can act as oxidants in photocatalytic cycles, furnishing the desired alkoxy radicals. Several organic dyes have been exploited in these processes, including eosin Y (**OD13**) [163] and acridine red [164]. Wang and co-workers relied on this approach for the development of an organophotocatalytic vinylation of tetrahydrofuran derivatives with alkynes (Scheme 40) [165]. In this method, the photoexcited **OD13** induces the cleavage of the weak O–O bond of *tert*-butyl peroxide (**40.1**), generating a hydroxy radical and a *tert*-butoxy radical. The latter promotes an H abstraction from the substrate tetrahydrofuran (**8.1)**, giving access to an α-oxy C(sp^3^) radical, which is trapped by an alkyne **40.2**, providing the desired vinylation product **40.3**.

### Sulfur-centered radicals

#### Thiyl (sulfenyl) radicals

**Scheme 41:**
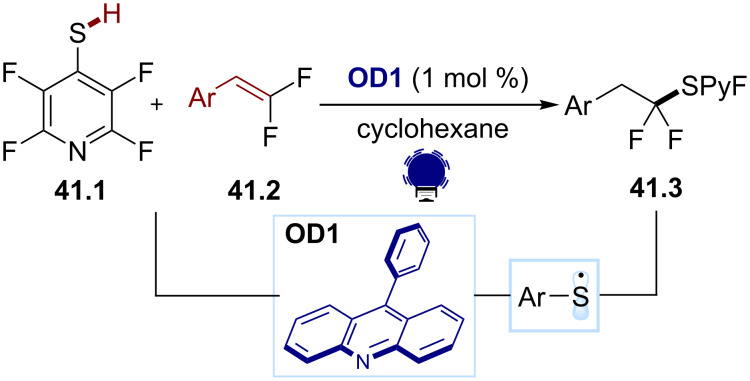
Illustrative example for the oxidative photocatalytic generation of thiyl radicals from thiols: the thiol-ene reaction with β-difluorostyrenes reported by Dilman and co-workers [168].

Thiyl radicals are common, versatile, strong nucleophilic radicals. They are efficient at performing atom abstractions, in particular with H-atoms, adding to π-systems and electrophiles, such as carbonyl compounds [166]. They can be generated from the UV irradiation of disulfides, sulfides or even thiols [167]. However, their use in organophotocatalysis is scarce. In substoichiometric quantities, they are efficient H atom shuttles and play a primordial role in hydrogen transfer mechanisms. For this reason, one of their major applications is the HAT to the cocatalyst (Scheme 3 and Scheme 9) [41,60]. In stoichiometric amounts, they generally add efficiently to π-systems and can be applied in thiol-ene reactions. Recently, Dilman and co-workers published a hydrosulfenylation of the β-difluorostyrenes **41.2** for the synthesis of the thioethers **41.3** using 9-PhAcr (**OD1**) as a photocatalyst (Scheme 41) [168]. The formed thioethers **41.3** could then be used as a *gem*-difluoroalkyl radical source for further transformations. Interestingly, 9-PhAcr (**OD1**) can only act as a photocatalyst in its protonated form. The thiol **41.1** acts as a proton source for **OD1**, allowing it to undergo a photoexcitation, leading to the excited state that can then oxidize the thiolate and generate the key S-centered radical. The latter then adds to the styrene. Other reports describe the use of phenyl glyoxylic acid [169] or eosin Y (**OD13**) for thiol-ene [170] and thiol-yne reactions [171].

#### Sulfonyl radicals

**Scheme 42:**
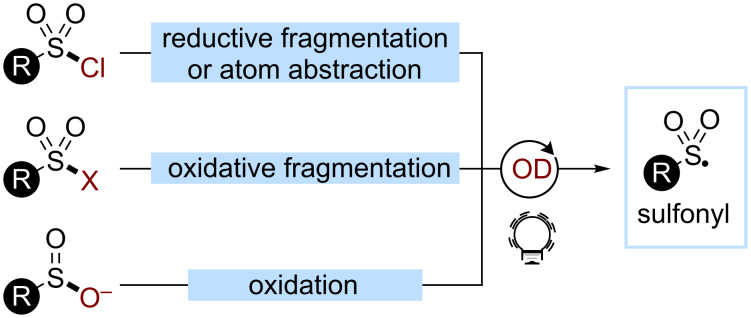
Main strategies and reagents for the generation of sulfonyl radicals used in organophotocatalysis.

Sulfonyl radicals show a good reactivity with π-systems, allowing the synthesis of nonsymmetrical sulfones [172]. However, they are also prone to fragmentations, resulting in the desulfonylation of the substrate and the generation of the corresponding C-centered radical. Sulfonyl radicals can be obtained from the fragmentation of sulfonic acid analogues, such as sulfonyl chlorides or sulfonyl hydrazides under reductive or oxidative conditions. Sulfinic acids can also be readily oxidized in the presence of a base, affording the desired sulfonyl radical (Scheme 42).

**Scheme 43:**
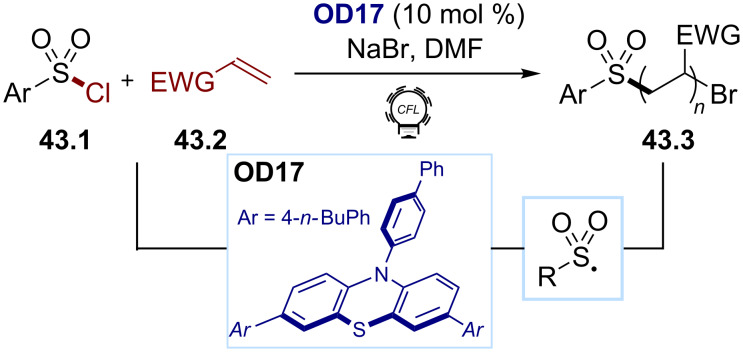
Illustrative example for the reductive photocatalytic generation of sulfonyl radicals from arylsulfonyl chlorides: the acrylate and acrylamide polymerization reported by Chen and co-workers [173].

The fragmentation of sulfonyl chloride derivatives requires relatively strong reductive conditions. Most reductions occur at −1.30 V or lower values (with the exception of CF_3_SO_2_Cl: *E*_red_ = –0.18 V). Organophotocatalysis has been exploited to promote this reduction. For example, Chen and co-workers designed the new organic dye **OD17** in 2018 (Scheme 43, *E*(PC^•+^/PC*) ≈ −1.9 V), able of reducing the arylsulfonyl chlorides **43.1** (*E*_red_ = −0.94 V) to initiate the synthesis of the polyacrylates and polyacrylamides **43.3** [173].

**Scheme 44:**
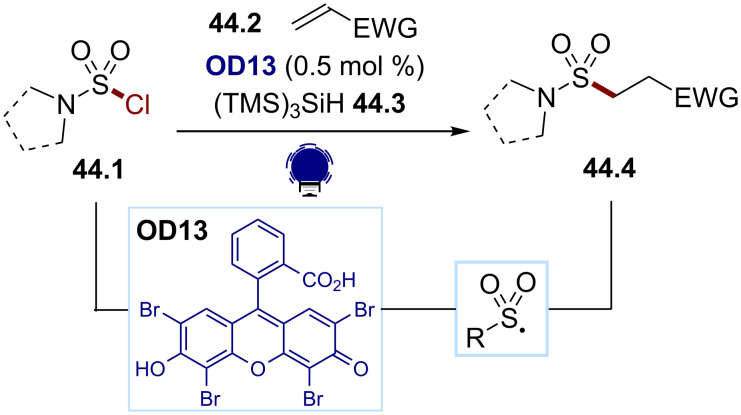
Illustrative example of a Cl atom abstraction strategy for the photocatalytic generation of sulfamoyl radicals from sulfamoyl chlorides: the radical chlorine abstraction for a Giese reaction reported by Gouverneur and co-workers [174].

To bypass the strong reductive conditions communally used for the activation of sulfamoyl chlorides to form the corresponding sulfamoyl radicals, Gouverneur and co-workers developed an efficient method based on chloride abstractions. They could successfully convert the sulfamoyl chlorides **44.1** to the alkyl sulfonamides **44.4** using eosin Y (**OD13**) as a photocatalyst and the supersilane **44.3** for a halogen abstraction (Scheme 44) [174]. Under visible-light irradiation, the excited state OD13* can oxidize the silane, generating, after deprotonation, a silyl radical, which can efficiently abstract the chlorine atom, resulting in the nucleophilic sulfamoyl radical. The latter can then undergo a Giese addition to an electron-deficient alkene **44.2**, affording the desired alkyl sulfonamides **44.4**.

**Scheme 45:**
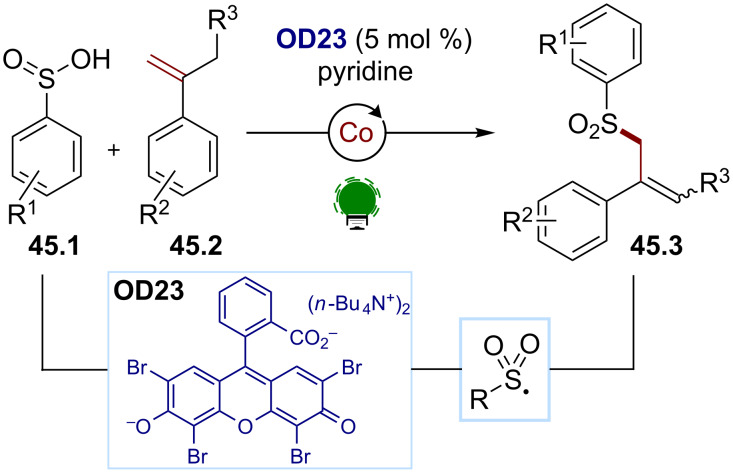
Illustrative example for the oxidative photocatalytic generation of sulfonyl radicals from sulfinic acids: the metallaphotoredox allyl sulfone synthesis reported by Lei and co-workers [178].

The SET oxidation of sulfinic acids and sulfinates for the generation of sulfonyl radicals has been thoroughly explored over the past decades [172]. These oxidations occur under mildly oxidative conditions, and eosin Y (**OD13**) stands out as an efficient photocatalyst for these steps [175–177]. This strategy is also compatible with transition metal catalysis, as shown by Lei and co-workers with an organophotocatalyzed radical cross-coupling method using the aryl sulfinic acids **45.1** and the styrenes **45.2** to generate the allyl sulfones **45.3** (Scheme 45) [178]. Under visible-light irradiation, the aryl sulfinic acid **45.1** is oxidized by the excited-state photocatalyst, a bis(tetrabutylammonium) salt of eosin Y, **OD23**, generating the desired sulfonyl radical. The latter adds to the styrene **45.2**, and the formed benzylic radical is intercepted by the cobalt catalyst, which can promote a dehydrogenation to form the allyl sulfone **45.3**. The colbalt cocatalyst secures the turnover of the photocatalyst by reducing it back to its ground state.

### Excited reactive intermediates (energy transfer)

In addition to electron transfer and atom transfer, EnT is one of the key modes of activation employed in photocatalysis. In order to act as a good energy transfer catalyst, an organic molecule, upon light irradiation, should undergo a productive intersystem crossing (ISC) and reach an electronically excited triplet state. The latter can transfer its energy to a ground-state substrate in a process named “sensitization”. The so-formed excited substrate is a valuable reactive intermediate for achieving various transformations and structural modifications. This approach has been widely exploited to generate singlet O_2_, a versatile oxidant often applied in organic synthesis [179].

**Scheme 46:**
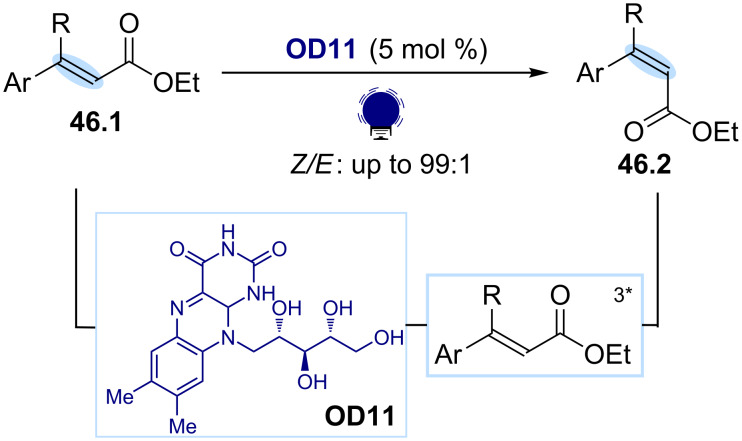
Illustrative example for the photocatalytic generation of electronically excited triplet states: the *E*-to-*Z* isomerization of olefins reported by Gilmour and co-workers [180].

Additionally, energy sensitization has emerged has a powerful strategy for promoting the contrathermodynamic *E*-to-*Z* isomerization of olefins. Inspired by the mechanism of vision in mammals, which implies a triplet state-mediated *E*-to-*Z* isomerization of retinal, Gilmour and co-workers reported an organophotocatalytic method for the isomerization of the α,β-unsaturated esters **46.1** (Scheme 46) [180]. In this protocol, (−)-riboflavin (**OD11**) is exploited as an organic photocatalyst, capable of absorbing light, transferring its triplet energy to the *E*-substrate and triggering the isomerization of the double bond with a high level of selectivity.

Recently, the same group extended this strategy to the geometrical isomerization of several classes of substrates, including β-ionyl derivatives [181], alkenylsilanes [182], and vinyl phosphonates [183]. In addition to riboflavin, other organic dyes, such as benzophenone, anthracene, 2-iodo-9-fluorenone [184], and carbazole- and cyanobenzene-based organophotocatalysts [185] have been successfully employed as photosensitizers for these isomerizations.

**Scheme 47:**
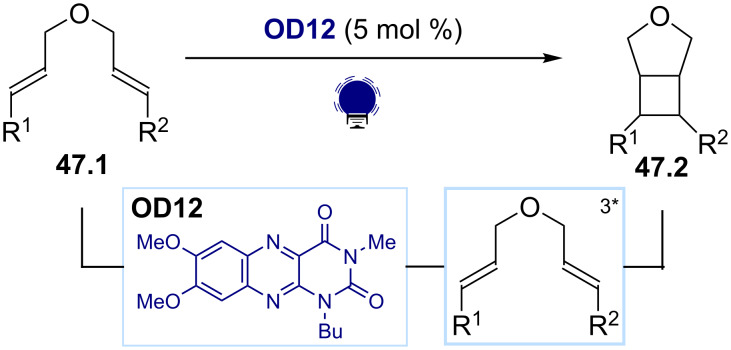
Illustrative example for the photocatalytic generation of electronically excited triplet states: the [2 + 2] cycloaddition of dienes reported by Chibulka and co-workers [188].

Another application of photosensitization is related to the development of thermally forbidden [2 + 2] cycloadditions. These reactions can be promoted by the direct excitation of the substrate, generally using UV light. However, the key triplet state intermediate can also be accessed by the energy transfer from a suitable photosensitizer. Seminal reports by Bach [186] and Sivaguru [187] demonstrated that thioxanthone-based organic dyes can promote these transformations. It has been shown that flavin-based dyes can also act as photosensitizers for this reaction (Scheme 47) [188]. In particular, alloxazine (**OD12**), which is structurally similar to flavin, has been used for promoting an intramolecular [2 + 2] cycloaddition of the dienes **47.1** to form the cyclobutane **47.2**.

## Conclusion

Organic dyes have emerged as efficient, cheap, and sustainable catalysts for photochemical transformations. In the last years, new scaffolds offering valuable alternatives to the established transition metal-based photocatalysts have appeared.

The structures of these light-absorbing molecules can be tuned, improving their photophysical properties and broadening their field of application. This has allowed the development of various organophotocatalytic methods, involving photoredox, energy, or atom transfer steps for accessing key reactive intermediates, including both open-shell species and excited-state molecules. In this review, we provided an overview on these reactive intermediates, and how they can be formed by using organic dyes as the photocatalysts. Each approach was illustrated by a few selected examples, with no effort to be exhaustive. Organic dyes have demonstrated a compatibility with other catalytic processes, such as metal catalysis and organocatalysis, further extending the possibilities for bond formations [29,189]. Furthermore, these scaffolds are invariably less toxic than their inorganic counterparts, rendering them available for an application in biological systems [155,190], and promising developments are also expected in emerging fields, such as photoelectrochemistry [84,191–192].

We believe that organophotocatalysis has consolidated its foundation and will play an increasingly important role in the development of novel photochemical transformations. This conceptual review summarizing the state-of-the art for the generation of reactive intermediates using organic dyes, shows the impressive progress that has already been realized in the last decade, and will give a first insight in the field for general synthetic chemists.
